# Personalized health risk assessment based on single-cell RNA sequencing analysis of a male with 45, X/48, XYYY karyotype

**DOI:** 10.1038/s41598-022-25308-w

**Published:** 2022-12-02

**Authors:** Magdalena Koczkowska, Marcin Jąkalski, Dorota Birkholz-Walerzak, Anna Kostecka, Mariola Iliszko, Magdalena Wójcik, Krzysztof Lewandowski, Katarzyna Milska-Musa, Patrick G. Buckley, Kinga Drężek, Ulana Juhas, Ewa Kuziemska, Agnieszka Maciejewska, Ryszard Pawłowski, Bartosz Wasąg, Natalia Filipowicz, Katarzyna Chojnowska, Urszula Ławrynowicz, Jan P. Dumanski, Beata S. Lipska-Ziętkiewicz, Jakub Mieczkowski, Arkadiusz Piotrowski

**Affiliations:** 1 Faculty of Pharmacy, Medical University of Gdansk, Gdansk, Poland; 2 3P-Medicine Laboratory, Medical University of Gdansk, Gdansk, Poland; 3 Department of Paediatrics, Diabetology and Endocrinology, Medical University of Gdansk, Gdansk, Poland; 4 Department of Biology and Medical Genetics, Medical University of Gdansk, Gdansk, Poland; 5 Laboratory of Clinical Genetics, University Clinical Centre, Gdansk, Poland; 6 Department of Laboratory Medicine, Medical University of Gdansk, Gdansk, Poland; 7 Department of Quality of Life Research, Faculty of Health Sciences With the Institute of Maritime and Tropical Medicine, Medical University of Gdansk, Gdansk, Poland; 8 Genomics Centre, Genuity Science, Dublin, Ireland; 9 Department of Forensic Medicine, Medical University of Gdansk, Gdansk, Poland; 10 Department of Immunology, Genetics and Pathology and Science for Life Laboratory, Uppsala University, Uppsala, Sweden; 11 Rare Diseases Centre, Medical University of Gdansk, Gdansk, Poland; 12 Clinical Genetics Unit, Department of Biology and Medical Genetics, Medical University of Gdansk, Gdansk, Poland

**Keywords:** Cancer genetics, Cytogenetics, Gene expression, Medical genetics, Sequencing

## Abstract

Numeric sex chromosome abnormalities are commonly associated with an increased cancer risk. Here, we report a 14-year-old boy with a rare mosaic 45, X/48, XYYY karyotype presenting with subtle dysmorphic features and relative height deficiency, requiring growth hormone therapy. As only 12 postnatal cases have been described so far with very limited follow-up data, to assess the proband’s long-term prognosis, including cancer risk, we performed high-throughput single-cell RNA sequencing (scRNA-seq) analysis. Although comprehensive cytogenetic analysis showed seemingly near perfect balance between 45, X and 48, XYYY cell populations, scRNA-seq revealed widespread differences in genotype distribution among immune cell fractions, specifically in monocytes, B- and T-cells. These results were confirmed at DNA level by digital-droplet PCR on flow-sorted immune cell types. Furthermore, deregulation of predominantly autosomal genes was observed, including *TCL1A* overexpression in 45, X B-lymphocytes and other known genes associated with hematological malignancies. Together with the standard hematological results, showing increased fractions of monocytes and CD4+/CD8+T lymphocytes ratio, long-term personalized hemato-oncological surveillance was recommended in the reported patient.

## Introduction

According to EUROCAT, the European network of population-based congenital anomaly registries, chromosomal abnormalities occur in 43.6/10,000 live births with trisomies 21, 18 and 13, and the sex chromosomes abnormalities being the most common^1^. The prevalence of males with extra Y chromosomes, such as 47, XYY, 48, XYYY or 49, XYYYY, is much lower, but is difficult to determine it precisely as these subjects might appear phenotypically healthy until fertility issues arise in adulthood. The first 48, XYYY case was described over 50 years ago^2^ and only 11 additional individuals have been reported to date, including five mosaic (Table S1). Here, we report a 14-year-old boy presenting moderate height deficiency and mild dysmorphic features with a mosaic 45, X/48, XYYY karyotype. We performed a comprehensive cytogenetic and molecular analysis to better understand the mechanism behind this rare disorder and to provide the individual as well as his family with reliable information regarding his specific risk for clinical/cancer complications and therapeutic perspectives.

### Clinical presentation

**Figure 1:**
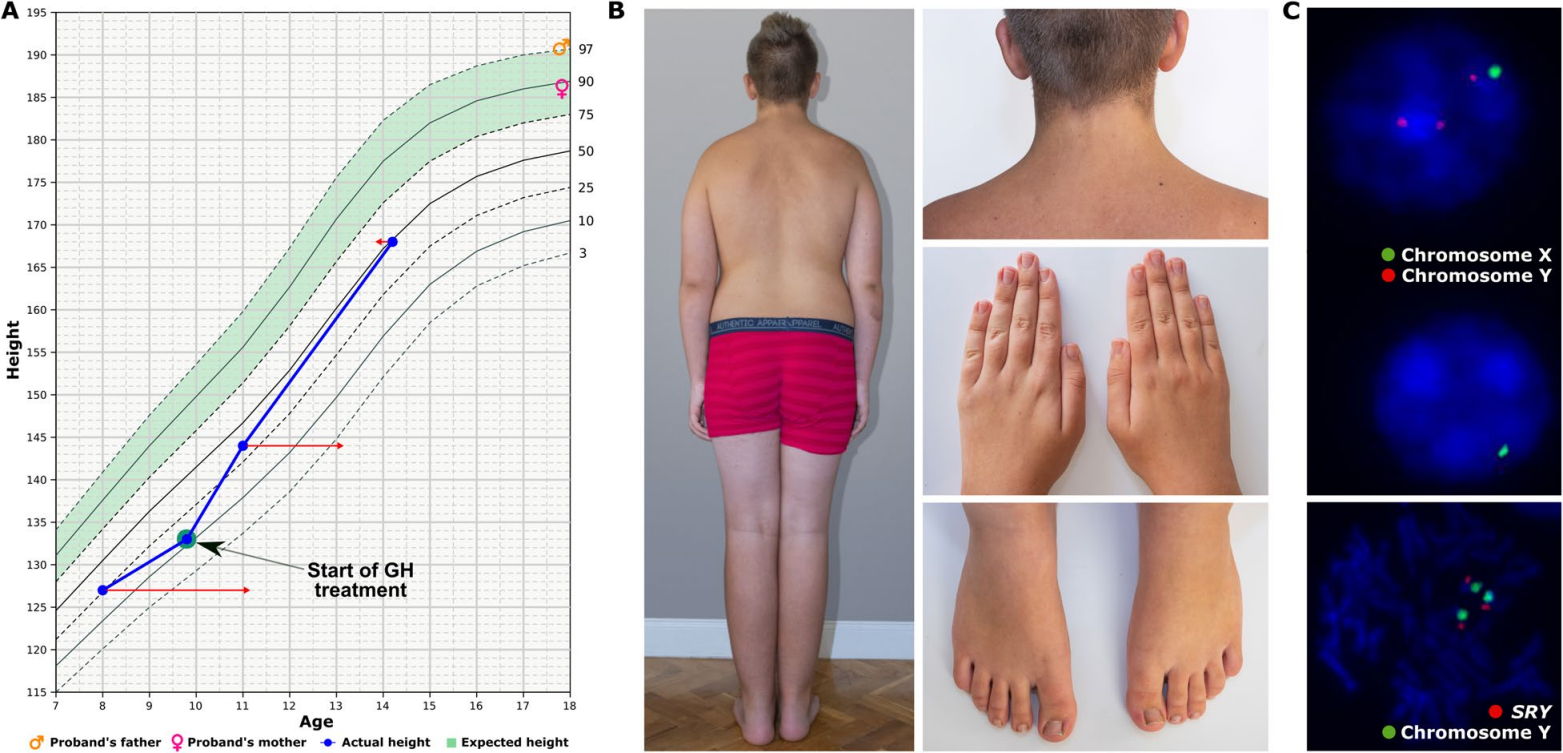
Clinical diagnostics overview. Panel (**A**) shows the proband’s growth chart over 6 years (blue line).^22^ At 8 years of age, the proband’s height was 127 cm (25th percentile) with the standard deviation score (SDS) of -0.6. The individual’s mother and father height are at 90th and 97th percentiles, respectively, with mid parental height of + 1.8 SDS (the green zone presents the proband’s expected height range). Growth hormone (GH) therapy was implemented successfully two years later, resulting in the patient’s height increase to the 50th percentile at the time of the most recent follow-up at 14 years of age. The red arrows indicate the corresponding bone age at the time of follow-up visits. At 8 years of age the proband had significantly accelerated bone maturity (bone age corresponding to 11 years) with the gradual decrease to 13 years at the age of 11 and the bone age appropriate for chronological age at the time of the most recent follow-up at 14 years old. Panel (**B**) shows images of short wide neck with low “M-shaped” hairline and hypoplastic nails, clinical features characteristic for Turner syndrome (45, X). Other subtle dysmorphic features included low-set ears, buckle-shaped chest, widely spaced breast nipples and numerous pigmented marks (not shown). Panel (**C**) presents standard cytogenetics results of the proband’s peripheral blood lymphocytes by fluorescent in situ hybridization (FISH) analyses, resulting in the 45,X/48,XYYY karyotype confirmation, i.e. nuc ish(DXZ1 × 1)[55]/(DXZ11, DYZ × 3)[62], with the normal *SRY* locus. Details of cytogenetics results are described in the Supplementary Information.

The child was conceived through assisted reproductive technology due to parental asthenospermia (intrauterine insemination procedure with homologous father’s semen). Born at term after an uneventful pregnancy with birth weight 4850 g and Apgar score 10, had psychomotor development in infancy and early childhood within the normal range. At the age of 8 the individual was referred to an endocrinologist due to a slower than expected mid-parental height growth rate (Fig. 1A). On physical examination, abnormal body proportions (greater sitting height than standing) and several dysmorphic features albeit resembling his father were noted (Fig. 1B). The patient was overweight with body mass index at 90–97th percentile (21.4 kg/m^2^) and had significantly accelerated bone maturity. Ultrasound images of thyroid gland, abdominal organs, heart and testes (Tanner stage 1) were normal. Subsequently, on magnetic resonance imaging an empty sella with the preserved signal from the posterior pituitary was observed, which prompted growth hormone (GH) stimulating testing. Results of clonidine and glucagon tests confirmed the diagnosis of somatotropin hypopituitarism and consequently GH treatment was applied at a dose of 0.018 mg/kg. Primary adrenal insufficiency and other hormonal abnormalities were ruled out by normal synthetic ACTH test results, pre-pubertal gonadotropin response in the LHRH test, normal pituitary TSH reserve in the synthetic TRH test and normal results of oral glucose tolerance test. The indistinct clinical picture and presence of multiple dysmorphic features, albeit mild, prompted further genetic analysis leading to diagnosis of 45, X/48, XYYY (Fig. 1C). At the most recent follow-up at 14.5 years of age, the proband’s height is at the 50th percentile as a result of a good response to GH treatment, with bone age appropriate for chronological age, which significantly improves the prognosis of his final height. Physical sexual development is according to his age (Tanner stage 3), but the perception of sexual interest is low in psychological evaluation. Ultrasound images of testicles are normal (6.2 ml and 6.5 ml testicle volume, respectively). Serum gonadotropin and testosterone levels are appropriate to the stage of sexual maturation. The patient’s intellectual functioning is average, with a Full Scale IQ score of 100 and no differences between nonverbal and verbal IQ. Extended psychological evaluation revealed no behavioral disorders, but difficulties in spatial orientation.

## Methods

This study was approved by the Institutional Review Board and Ethics Committee at the Medical University of Gdansk (NKBBN/215/2020) and all methods were carried out in accordance with relevant guidelines and regulations. The individual’s parent(s) provided written informed consent prior to the start of the study. Whole blood, buccal swabs and/or fibroblasts were collected from the proband and his father. Fluorescence-activated cell sorting (FACS) and DNA extraction were performed in accordance with the manufacturers’ protocols (details in “Samples collection and sorting of blood cell with fluorescence-activated cell sorting (FACS)” and “DNA extraction” in the Supplementary Information). The initial genetic work-up included conventional karyotyping and fluorescent in situ hybridization (FISH) analyses of lymphocytes and cultured skin fibroblasts, followed by chromosomal microarray analysis (CMA), chromosome Y short-tandem repeat (Y-STR) haplotyping and digital droplet PCR (ddPCR) of FACS-sorted cells (details in “Cytogenetic and molecular studies” in the Supplementary Information). To assess the risk of hematological complications the standard diagnostic analysis was performed, including a complete blood count, microscopy and a flow cytometry immunophenotyping. Single-cell RNA sequencing analysis (scRNA-seq) was performed on freshly isolated peripheral blood mononuclear cells (PBMCs) according to the manufacturer’s protocol and raw data analysis was done with the CellRanger software (10xGenomics). Further data processing was performed in R and Seurat to normalize, cluster and visualize the scRNA-seq data, and to identify cells with or without chromosome Y^3^. All statistical analysis were done in R computational environment. SingleR, followed by manual curation, was used to label the individual immune cell types. Differential gene expression with integration and comparison against healthy male controls was again done in R and Seurat. All methodological details related to the scRNA-seq analysis are provided in the following sections of the Supplementary Information, i.e. “scRNA-seq analysis”, “Processing of scRNA-seq data”, “Estimating mosaicism at single-cell level”, “Comparison against public PBMC data” and “Differential expression analyses”.

## Results

**Figure 2:**
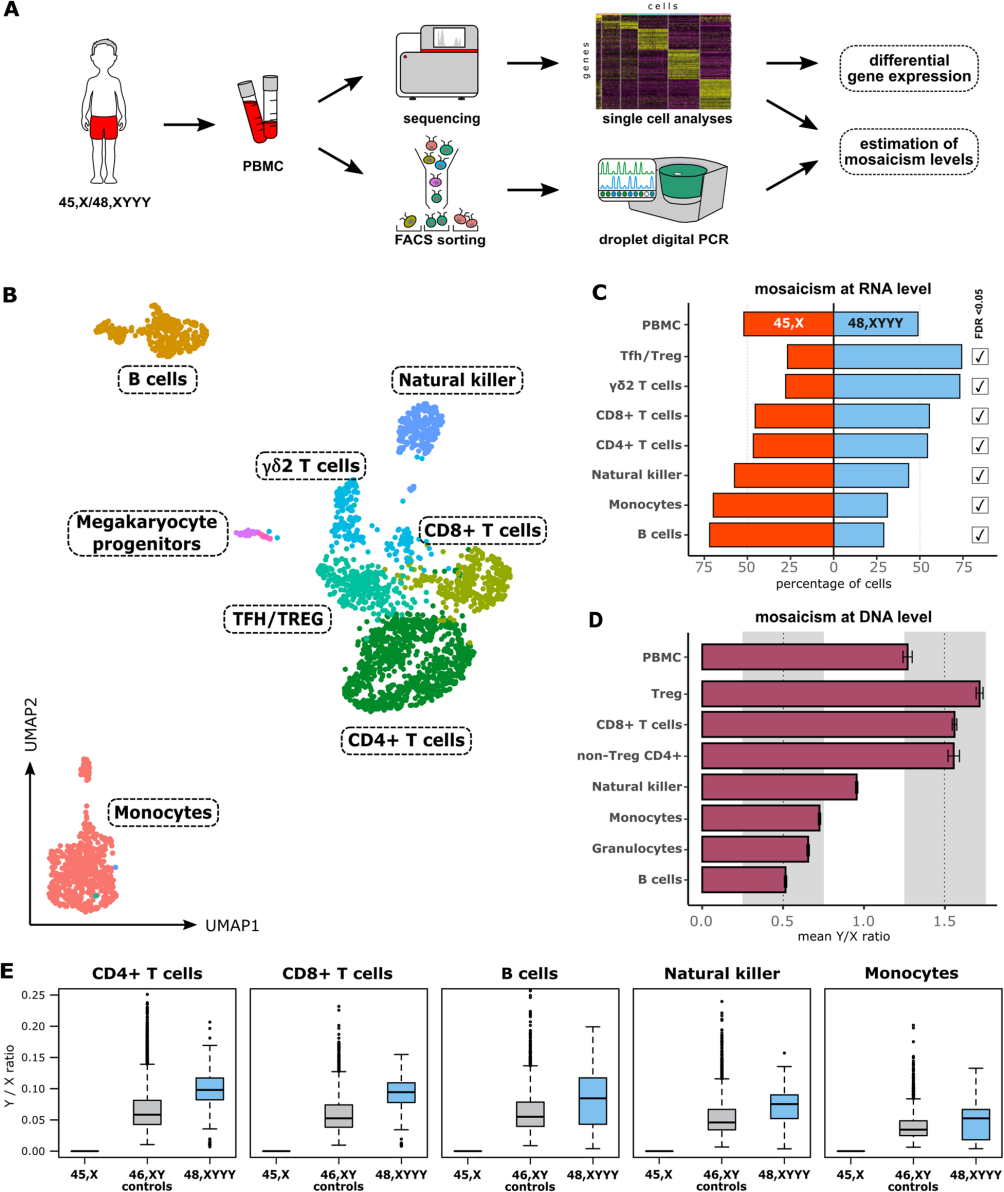
Summary of single-cell RNA sequencing analysis. Panel (**A**) shows a schematic overview of the experimental design. Panel (**B**) shows two-dimensional UMAP projection of the single-cell RNA sequencing (scRNA-seq) data. Eight main clusters of immune cells are colored according to their inferred cell type. Each colored dot corresponds to a single cell. Panel (**C**) shows distribution of the two mosaic populations 45, X (red) and 48, XYYY (blue) in the total sample of peripheral blood mononuclear cells (PBMC) and in the selected major subpopulations at single cell level. Expression of any single Y-linked gene was used to assign cells to the 48, XYYY fraction. The checkboxes on the right side of the plot indicate whether the differences of a given cell type between 45, X and 48, XYYY cells are significant (FDR < 0.05, *p* values compute with Monte Carlo permutation test) and are not an effect of random sampling (details in Table S12 in the Supplementary Information). Panel (**D**) shows mosaicism level assessment by digital droplet PCR (ddPCR) using the AMELX/AMELY assay. Individual’s fractions of the FACS-sorted cells stay in agreement with the immune cell fractions from scRNA-seq in terms of the ratios of Y and X-containing subpopulations. Panel (**E**) shows ratios of the Y-linked to X-linked expression in the 48, XYYY cell population as compared to the public PBMC data of healthy males. Labels on the X axis correspond to the IDs of donors used in the public datasets or their age (for *GSE128879*). In each of the graphs the 48, XYYY fraction of the studied individual’s PBMC displays a much higher Y/X ratio than the males used for comparison (details in Fig. S7).

**Figure 3:**
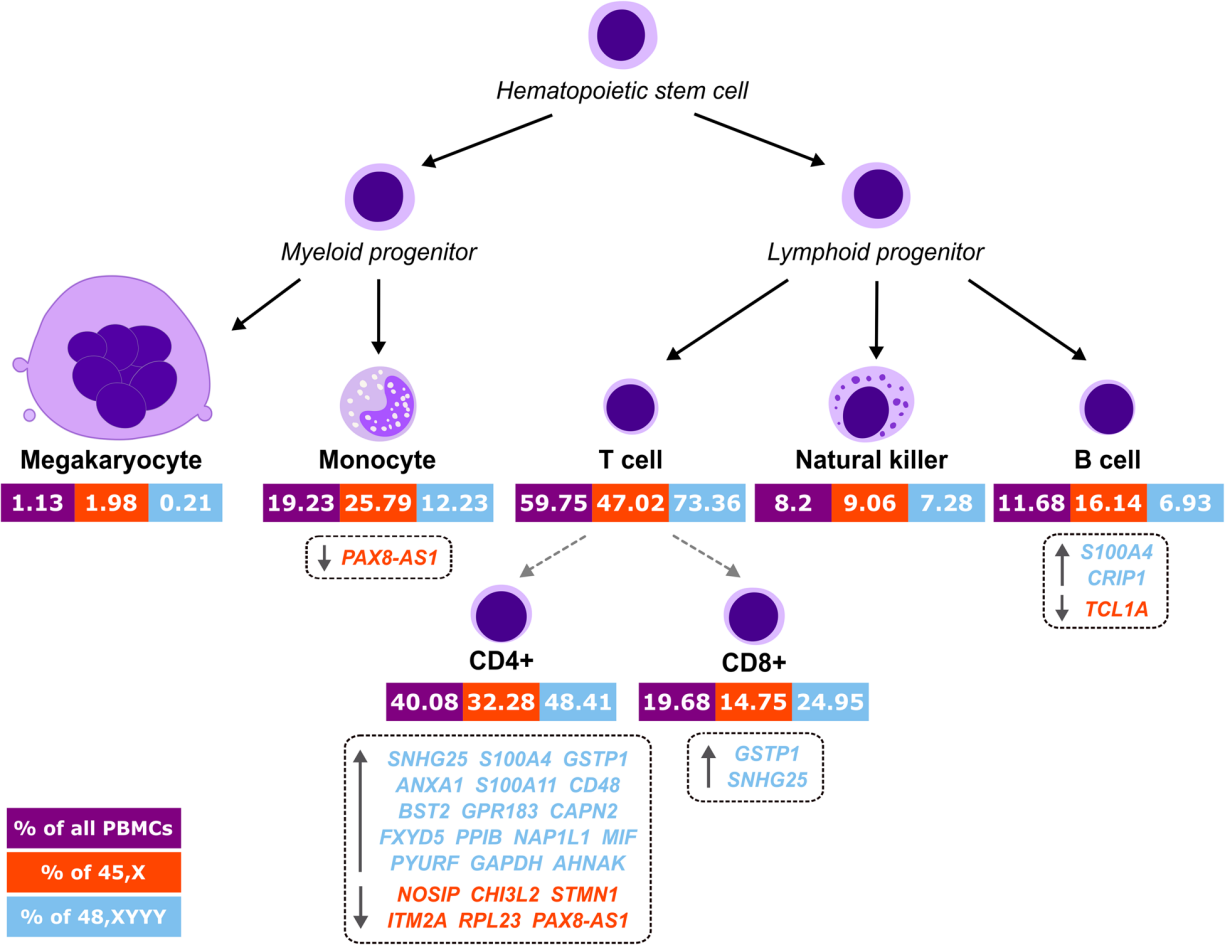
Hematopoiesis scheme with the mosaic 45, X and 48, XYYY cell population fractions and the list of differentially expressed genes. Summarized statistics of the mosaic 45, X and 48, XYYY cell populations are overlaid on the hematopoiesis scheme. Only the main identified cell types are shown. Values shown in the purple, red, and blue boxes correspond to the total number or fractions (%) of single cells among all studied cells, 45, X cells only or 48, XYYY cells only, respectively. Solid black arrows follow the main developmental directions of blood cells. Dashed grey arrows correspond to main paths of lymphopoiesis of different T lymphocytes. List of genes is shown as dashed boxes with up and down arrows presenting the direction of gene dysregulation.

Cytogenetic analysis revealed the presence of a mosaic 45, X/48, XYYY karyotype with mosaicism proportion of 44–62% for 48, XYYY cell population (Figs. 1C, S1). Normal karyotypes were reported in parents’ peripheral blood; the father had also a normal karyotype in fibroblasts (Fig. S1). No additional genomic imbalances were observed in the proband’s karyotype by CMA and Y-STR haplotype analyses (Figs. S2, S3). In terms of scRNA-seq, the total number of good quality cells was 2936 with a median of 2066 genes and 7400 UMI counts per cell (Tables S2, S3). Expression-based clustering and visualization of the data in a two-dimensional space yielded twelve distinct clusters representing eight major types of immune cells (Figs. 2A–B, S4, Tables S4, S5), reflecting physiological proportions seen in PBMCs. The status of each individual cell (45, X or 48, XYYY) was determined by detecting the presence or a complete absence of Y-linked expression (Fig. S5 and Table S6). In total, the cells with and without chromosome Y constituted 48.43% and 51.57%, respectively (Table S7). This nearly perfect balance was no longer observed when analyzing individual immune cell fractions, i.e. T-cell populations were mostly composed of 48, XYYY cells, while B cells and monocytes of 45,X cells. These results were confirmed by an independent molecular method, i.e. ddPCR on FACS-sorted cells (Figs. 2C–D, 3 and Table S8). The high frequencies of triple Y cells were accompanied with significantly higher expression from Y chromosome genes (Figs. 2E, S6), with a lower Y/X ratio observed in PBMCs of healthy male controls (Fig. S7). Comparison of gene expression between the major immune cell populations with 45, X and 48, XYYY karyotypes revealed a list of 31 genes whose expression differed significantly (DEGs) (Wilcoxon rank sum test with a Bonferroni-adjusted *p* value < 0.05), including 13 genes potentially related to cancer/hematological complications (Fig. 3, Tables S9, S10). Hematological profile was within normal range, except for a borderline value of classical monocytes (91.3%) and an increased ratio (2.6:1) of CD4+ versus CD8+ T lymphocytes (Fig. S8 and Table S11).

## Discussion

We report here the first comprehensive molecular analysis of a pre-pubertal individual with a rare mosaic 48, XYYY karyotype. Because only 12 postnatal cases have been described so far with limited follow-up data, the individual’s long-term prognosis, including cancer risk, was difficult to predict. The combination of cytogenetic and scRNA-seq analyses along with the detailed clinical information resulted in providing personalized care to the patient and his family.

Sex chromosome aneuploidy may affect physical, developmental, behavioral and cognitive functioning, with each extra chromosome increasing the severity^4^, in line with the previous 48, XYYY case reports (Table S1). In contrast to these observations, the individual described in this study did not have gross birth defects, a rather normal psychomotor development and intellectual functioning. The genetic diagnosis was prompted by the presence of very subtle dysmorphic features and moderate height deficiency, signs not considered to be cardinal for the Y chromosome polysomy.

There are two well described mechanisms underlying gains and losses of whole chromosomes, i.e. mitotic or meiotic nondisjunction, when chromosomes do not properly separate and one daughter cell miss a chromosome that may be gained by the other daughter cell, and, less commonly, structural chromosomal abnormalities that might result in aneuploidy as well. We likely excluded the possibility that the proband carries other structural unbalanced rearrangements or copy number neutral loss-of-heterozygosity by performing chromosomal SNP microarray analysis (Fig. S2). Hence, nondisjunction(s) appear(s) to be the more likely explanation. In the case of the double Y chromosomes (47, XYY), the nondisjunction could readily explain this abnormality, but this is not explanatory for 48, XYYY. Moreover, we cannot exclude the possibility of conceiving a normal ovum by a YYY-sperm that was a result of errors in spermatogenesis through nondisjunction or anaphase lagging. It is noteworthy that the proband’s father had normal karyotype in peripheral blood and fibroblasts, analyzed by two cytogenetic techniques, i.e. the classical GTG-banding karyotyping at the 550-band stage of resolution and FISH with a much higher resolution (Fig. S1). In order to exclude the latter potential mechanism, the father’s testicular tissue biopsy should have been performed as this tissue could be mosaic. However, in line with the research ethics protocols, there was no indications for performing such an invasive medical procedure. Furthermore, gonosomal mosaicism of the father for the 48, XYYY genotype does not explain presence of 45, X lineage in the proband. Yet another possible explanation is a series of nondisjunction of the Y chromosome in a normal zygote. However, in that scenario we should have detected at least four cell lineages: 45, X, 46, XY, 47, XYY and 48, XYYY that originated via nondisjunction in the course of subsequent cellular divisions. As we showed in Figs. 1C, S1, only 45, X and 48, XYYY cell lines were identified in the reported patient. Hence, the 46, XY and 47, XYY should have been eliminated during the ontogenesis or might be present in other tissues/organs that were not sampled. Altogether, due to the very strong speculative nature, these potential mechanism(s) needs to be interpreted with caution.

As the proband has a mosaic karyotype with a 45, X cell lineage and, considering the increased risk for gonadal tumors in women with Turner syndrome and a Y mosaic genotype^5^, the relevant question is whether the bilateral prophylactic gonadectomy applies. Evaluation of clinical guidelines for timing of gonadectomy shows substantial inconsistencies. Current recommendations advocate individualized and conservative approach by taking into account specific location of the gonads (scrotal or non-scrotal), the internal and external phenotype and sex of rearing^6^. GH therapy, usually implemented in women with Turner syndrome, is another risk factor of neoplasia development^7^, thus the pros and cons should be considered before going ahead with the treatment.

Loss of chromosome Y (LOY) in blood of ageing men has been correlated with shorter survival and higher risk of non-hematological cancer mortality^8^. Several case reports of 47,XYY males also demonstrate the increased risk for various neoplasms, mostly for hematological malignancies^9–14^. Notably, large epidemiological study by Jorgensen et al.^15^ on 82 individuals with 47, XYY syndrome did not confirm these observations, but the median age of the studied cohort was only 12.7 years old. Considering the occurrence of these specific cancer risk factors, including, but not limited to gonadal tumors, and the ongoing GH therapy, it was crucial from the clinical perspective to evaluate the overall cancer risk in the individual described here.

Single-cell sequencing provided a powerful personalized medicine approach to tailor the clinical management and targeted therapy^16^. The scRNA-seq transcriptomic analysis performed in this study confirmed the equal proportions of 45, X and 48, XYYY cell lineages in a PBMC sample, in line with the standard cytogenetics results (details in Supplementary Information). However, we demonstrated that these proportions differ between the individual’s specific immune cell fractions, with up to two times greater fold change for monocytes, B- and T-cells (Figs. 2, 3 and Table S12). These findings suggest that the numerical Y chromosome abnormalities likely have an effect on hematopoietic cells, and should not be considered as a neutral event. While several studies show the association between LOY and aberrant clonal expansion of cells^17,18^, the role of an extra Y chromosome cannot be excluded. Comparative analysis against an independent cohort of healthy males revealed that the high frequencies of triple Y cells in proband were accompanied with significantly higher expression from the Y chromosome (Figs. 2E, S6). Nevertheless, further studies are required to fully understand the mechanism underlying the clonal expansion.

Detailed examination of the identified DEGs revealed dysregulation of a considerable number of autosomal genes (Fig. 3). Enrichment analysis performed to understand the biological significance of transcriptional changes showed that these genes are important for a range of physiological functions. Altogether they encode regulators of stem/progenitor cell commitment and bridges between innate and adaptive immune responses. Besides, as components of immunosurveillance machinery they are directly involved in T-cell signaling, which might provide a possible explanation for the frequency discrepancy of T lymphocytes between populations of 45, X and 48, XYYY cells (Table S13). Notably, 6/31 autosomal genes are associated with hematological malignancies (Table S10), including *TCL1A* (MIM * 186960), a known leukemia/lymphoma risk factor. The protein encoded by *TCL1A* acts as a co-activator of the cell survival kinase AKT with the overexpression usually observed in hematological malignancies of T and B cells^19^. Our findings demonstrate that *TCL1A* expression is dysregulated in CD19+B cells, in line with the previous studies^18,20^. However, the mechanisms of its regulation are largely unknown. GWAS studies reported recently that specific single nucleotide polymorphisms near *TCL1A* (rs2887399) might be associated with the predisposition to LOY^17,20,21^, but this genotype was not present in the proband (Fig. S2).

Given all evidence from the previous and the current studies, hematological surveillance of patients with numerical Y chromosome abnormalities is advisable. Although standard hematological analysis has not revealed now any significant abnormalities in the presented individual, except for increased fractions of monocytes and CD4+/CD8+T lymphocytes ratio that do require follow-up, it should be kept in mind that the proband is a 14-year-old boy before puberty, hence he was referred to periodic hematological evaluation, pending possible bone marrow biopsy.

In conclusion, specific hematological malignancy risk in GH-treated phenotypic males with 45, X/48, XYYY mosaicism is understudied and remains to be ascertained. Transcriptomic results presented here are concerning and point to the need for careful hematological surveillance in these patients. Obviously, a close follow-up of the testes, including regular clinical and sonographic evaluation and IGF-I evaluation while on GH treatment, is mandatory. As most adult males reported to date with 48, XYYY karyotype were diagnosed with azoospermia, likely due to decreased testicular function at the end of puberty, early fertility preservation strategies might also be considered. This study highlights the clear synergistic effects and clinical utility of close translational collaboration between the research laboratory and the clinic enabling genotype-driven personalized medicine.

## Supplementary Information

Supplementary Information.

## Data Availability

Raw, de-identified RNA-sequencing files were deposited in European Genome-Phenome Archive (https://ega-archive.org/): accession no. EGAS00001005697 (EGAD00001008645). The code used to process the data described in this study is available at: https://github.com/jakalssj3/45X_48XYYY.

## References

[1] Wellesley D, Dolk H, Boyd PA (2012). Rare chromosome abnormalities, prevalence and prenatal diagnosis rates from population-based congenital anomaly registers in Europe. Eur. J. Hum. Genet..

[2] Townes PL, Ziegler NA, Lenhard LW (1965). A patient with 48 chromosomes (XYYY). Lancet.

[3] Butler A, Hoffman P, Smibert P, Papalexi E, Satija R (2018). Integrating single-cell transcriptomic data across different conditions, technologies, and species. Nat. Biotechnol..

[4] Linden MG, Bender BG, Robinson A (1995). Sex chromosome tetrasomy and pentasomy. Pediatrics.

[5] Gravholt CH, Andersen NH, Conway GS (2017). Clinical practice guidelines for the care of girls and women with Turner syndrome: Proceedings from the 2016 cincinnati international turner syndrome meeting. Eur. J. Endocrinol..

[6] McCann-Crosby B, Mansouri R, Dietrich JE (2014). State of the art review in gonadal dysgenesis: Challenges in diagnosis and management. Int. J. Pediatr. Endocrinol..

[7] Gawrychowska A, Iżycka-Świeszewska E, Lipska-Ziętkiewicz BS (2020). Dysgerminoma with a somatic exon 17 KIT mutation and SHH pathway activation in a girl with Turner syndrome. Diagnostics.

[8] Forsberg LA, Rasi C, Malmqvist N (2014). Mosaic loss of chromosome Y in peripheral blood is associated with shorter survival and higher risk of cancer. Nat. Genet..

[9] Xiao H, Dadey B, Block AW, Han T, Sandberg AA (1991). Extra Y chromosome in chronic lymphoproliferative disorders. Cancer Genet Cytogenet..

[10] Taub JW, Ravindranath Y, Mohamed AN (1993). Acute lymphoblastic leukemia in a 46, X/47, XYY mosaic male: clonal origin of leukemia in the XY-bearing stem-cell line. Am. J. Dis. Child..

[11] Trivedi AH, Bakshi SR, Roy SK (1995). An XYY male with acute lymphoblastic leukemia. Cancer Genet..

[12] Oguma N, Shigeta C, Kamada N (1996). XYY male and hematologic malignancy. Cancer Genet. Cytogenet..

[13] Palanduz S, Aktan M, Ozturk S, Tutkan G, le Cef K, Pekcelen Y (1998). 47, XYY karyotype in acute myeloid leukemia. Cancer Genet..

[14] Jo HC, Lee SW, Jung HJ, Park JE (2016). Esthesioneuroblastoma in a boy with 47, XYY karyotype. Clin. Exp. Pediatr..

[15] Jorgensen IF, Russo F, Jensen AB (2019). Comorbidity landscape of the Danish patient population affected by chromosome abnormalities. Genet. Med..

[16] Kim D, Kobayashi T, Voisin B (2020). Targeted therapy guided by single-cell transcriptomic analysis in drug-induced hypersensitivity syndrome: A case report. Nature.

[17] Terao C, Momozawa Y, Ishigaki K (2019). GWAS of mosaic loss of chromosome Y highlights genetic effects on blood cell differentiation. Nat. Commun..

[18] Dumanski JP, Halvardson J, Davies H (2021). Immune cells lacking Y chromosome show dysregulation of autosomal gene expression. Cell Mol. Life Sci..

[19] Laine J, Kunstle G, Obata T, Sha M, Noguchi M (2000). The protooncogene *TCL1* is an Akt kinase coactivator. Mol. Cell.

[20] Thompson DJ, Genovese G, Halvardson J (2019). Genetic predisposition to mosaic Y chromosome loss in blood. Nature.

[21] Zhou W, Machiela MJ, Ishigaki K (2016). Mosaic loss of chromosome Y is associated with common variation near *TCL1A*. Nat. Genet..

[22] Kułaga Z, Różdżyńska-Świątkowska A, Palczewska I (2010). Siatki centylowe wysokości, masy ciała i wskaźnika masy ciała dzieci i młodzieży w Polsce - wyniki badania OLAF. Standardy medyczne/Pediatria.

