# Supplementary material for: Personalized health risk assessment based on single-cell RNA sequencing analysis of a male with 45, X/48, XYYY karyotype
Source: Sci Rep. 2022 Dec 2;12:20854. doi: 10.1038/s41598-022-25308-w (PMC9718746; doi:10.1038/s41598-022-25308-w)
Supplement: Supplementary file 1 — Supplementary Information. [file 41598_2022_25308_MOESM1_ESM.pdf]

Personalized health risk assessment based on single-cell RNA sequencing analysis of a male with 45,X/48,XYYY karyotype

Magdalena Koczkowska,[1,2,*] Marcin Jąkalski,[2,*] Dorota Birkholz-Walerzak,[3,*] Anna Kostecka,[1,2] Mariola Iliszko,[4,5] Magdalena Wójcik,[2] Krzysztof Lewandowski,[6] Katarzyna Milska-Musa,[7] Patrick G. Buckley,[8] Kinga Drężek,[2] Ulana Juhas,[2] Ewa Kuziemska,[4,5] Agnieszka Maciejewska,[9] Ryszard Pawłowski,[9] Bartosz Wasąg,[4,5] Natalia Filipowicz,[2] Katarzyna Chojnowska,[2] Urszula Ławrynowicz,[2] Jan P. Dumanski,[1,2,10] Beata S. Lipska-Ziętkiewicz,[11,12,#] Jakub Mieczkowski,[2,#] Arkadiusz Piotrowski,[1,2#]

[1] Faculty of Pharmacy, Medical University of Gdansk, Gdansk, Poland; [2] 3P-Medicine Laboratory, Medical University of Gdansk, Gdansk, Poland; [3] Department of Paediatrics, Diabetology and Endocrinology, Medical University of Gdansk, Gdansk, Poland; [4] Department of Biology and Medical Genetics, Medical University of Gdansk, Gdansk, Poland; [5] Laboratory of Clinical Genetics, University Clinical Centre, Gdansk, Poland; [6] Department of Laboratory Medicine, Medical University of Gdansk, Gdansk, Poland; [7] Department of Quality of Life Research, Faculty of Health Sciences with the Institute of Maritime and Tropical Medicine, Medical University of Gdansk, Gdansk, Poland; [8] Genomics Centre, Genuity Science, Dublin, Ireland; [9] Department of Forensic Medicine, Medical University of Gdansk, Gdansk, Poland; [10] Department of Immunology, Genetics and Pathology and Science for Life Laboratory, Uppsala University, Uppsala, Sweden; [11] Rare Diseases Centre, Medical University of Gdansk, Gdansk, Poland; [12] Clinical Genetics Unit, Department of Biology and Medical Genetics, Medical University of Gdansk, Gdansk, Poland.

[*] Drs. Koczkowska, Jąkalski and Birkholz-Walerzak contributed equally to the work.

# Drs. Lipska-Ziętkiewicz, Mieczkowski and Piotrowski contributed equally to the work.

Table of Contents

Supplementary Methods …………………………………………………………………………………… 3
Samples collection and sorting of blood cells with fluorescence-activated cell sorting (FACS) ……………. 3
DNA extraction ……………………………………………………………………………………………. 3
Cytogenetic and molecular studies ……………………………………………………………………….... 3
scRNA-seq analysis …………………………………………………………………………………………… 4
Processing of scRNA-seq data ………………………………………………………………………………… 4
Estimating mosaicism at single-cell level ………………………………………………………………….. 4
Comparison against public PBMC data ……………………………………………………………………… 5
Differential expression analyses ………………………………………………………………………….… 5
Supplementary Results ………………………………………………………………………………… 6
Detecting Y-linked expressions and estimating the level of mosaicism ……………………………………. 6
Supplementary Figures & Tables ……………………………………………………………………….... 7
Figure S1. Cytogenetics results from fibroblast cultures ………………………………………………… 7
Figure S2. Chromosomal microarray analysis (CMA) results with the *TCL1A* SNP (rs2887399) locus …... 8
Figure S3. Autosomal and Y-chromosome short-tandem repeat (Y-STR) DNA typing results………….. 9
Figure S4. Selected marker genes used to verify the computationally inferred cell clusters ………………… 10
Figure S5. Separation of the PBMC single cells into the two mosaic populations 45,X and 48,XYYY verified by two independent methods for processing UMI based single cell data ………………………………………. 11
Figure S6. Ratios of Y-linked to X-linked expression (normalized) in the 48,XYYY cell population in selected fractions of the immune cell types ……………………………………………………………………….. 11
Figure S7. Ratios of Y-linked to X-linked expression (normalized) in the 48,XYYY cell population as compared to the public PBMC data of healthy males ………………………………………………………………… 12
Figure S8. Flow cytometry immunophenotyping of monocytes and T lymphocytes in the blood of a proband as compared to a healthy control ……………………………………………………………………………… 13
Table S1. Clinical features of 14 cases reported as with pure or mosaic 48,XYYY karyotype …………… 14
Table S2. Summary of high throughput sequencing and Cell Ranger results ……………………………… 17
Table S3. Summary of quality filtering steps within Seurat ………………………………………………… 17
Table S4. Summary of clustering with Seurat ……………………………………………………………. 17
Table S5. Clusters calculated with Seurat by applying the default Louvain algorithm and their labels …….. 18
Table S6. Genes from chromosome Y identified in the studied sample ……………………………………… 19
Table S7. Summary statistics of 45,X and 48,XYYY cells identified among individual clusters and the entire sample ……………………………………………………………………………………………………….. 20
Table S8. Results of digital droplet PCR (ddPCR) assay …………………………………………………… 20
Table S9. List of 31 differentially expressed genes between 45,X and 48,XYYY populations ……………... 21
Table S10. Description of selected genes potentially related to cancer/hematological complications ……. 22
Table S11. Results of standard hematological analysis of the proband's peripheral blood ………………….23
Table S12. Details of Monte Carlo permutation test ………………………………………………………. 24
Table S13. Details of gene-set enrichment analysis ……………………………………………………….. 24
References …………………………………………………………………………………………………… 25

Supplementary Methods

Samples collection and sorting of blood cells with fluorescence-activated cell sorting (FACS)

Approximately ~12 ml of peripheral blood, fibroblasts and buccal swabs were collected from the individual and/or the individual's father for cytogenetic and molecular studies. Additional ~32 ml of whole blood was collected from an individual into BD Vacutainer® CPT™ Mononuclear Cell Preparation tubes (BD Biosciences) for the isolation of peripheral blood mononuclear cells (PBMCs) using density gradient centrifugation. PBMCs were either immediately processed for single-cell RNA sequencing analysis (scRNA-seq) and for FACS cell sorting or stored at -80ºC. FACS analysis was performed on FACS Aria III (Beckton Dickinson) and data were acquired and analyzed using BD FACSDiva™ Software (Becton Dickinson). The following individual lineages were isolated using the negative immunomagnetic separation (Miltenyi Biotec), i.e. CD4+ regulatory (TREG) and non - regulatory (non-TREG) T cells (based on markers CD4, CD25 and CD127), and positive immunomagnetic separation (Miltenyi Biotec), i.e. CD8+ T cell (defined as CD45+, CD3+, CD8+), B cells (defined as CD45+, CD3-, CD19+), Natural killer (NK) cells (defined as CD45+, CD3-, CD16+, CD56+), monocytes (defined as CD45+, CD3-, CD14+). Granulocytes were sorted independently.

DNA extraction

Genomic DNA was extracted from the whole blood and buccal swabs using DNA Blood Mid (QIAGEN) and PrepIT.L2P (DNA Genotek) kits, respectively, according to the manufacturers' instructions. The standard phenol-chloroform procedure was applied for DNA extraction from cultured skin fibroblasts and PBMCs. For the DNA extraction from sorted cells the *in-house* protocol with 1% N-Lauroylsarcosine sodium salt (Sigma) was applied. DNA quality and concentration was assessed with TapeStation 4150 (Agilent, Perlan Technologies) and Varioskan Lux (Thermo Fisher Scientific).

Cytogenetic and molecular studies

Conventional karyotyping was performed by GTG-banding at 550-band stage of resolution. Commercially available chromosome X and Y centromere probes (CCPX/Y probes from CytoTest) and a *SRY* specific probe (Cytocell) were used for fluorescent *in situ* hybridization (FISH) analyses. At least 10 metaphases and 100 interphase nuclei were evaluated, respectively. Semi-custom selected SNP array based on Global Screening Array Multiple Disease (Illumina) was used for genotyping study. The genotyping data were analyzed by Nexus Copy Number software version 6.0 (BioDiscovery). Chromosome Y short-tandem repeat (Y-STR) haplotyping was performed with two commercially available multiplex PCR kits: 1/ PowerPlex® ES17 Fast (Promega) for 16 autosomal polymorphic loci and Amelogenin used as a sex marker, and 2/ PowerPlex® Y23 Systems (Promega) for 23 Y chromosome polymorphic loci. The product's detection was carried out on ABI 3130 Genetic Analyzer (Applied Biosystems) and analyzed with GeneMapper ID v.3.2. Assessment of mosaicism level was performed using QX200 Droplet Digital PCR system (Bio-Rad Laboratories) with *AMELX/AMELY* TaqMan-based assay (Thermo Fisher Scientific).[1] All samples were run in duplicates, while the ratio *AMELY/AMELX* was analyzed using Bio-Rad's QuantaSoft (version 1.7.4.0917).

scRNA-seq analysis

Freshly collected and isolated PBMCs were processed for scRNA-seq analysis in line with the manufacturer's instructions. After cell number and viability counting using an automated EVE cell counter (NanoEnTek), the final sample was diluted in PBS with 0.04% bovine serum albumin to 10^6 cells/ml. The capturing and library construction were performed using the Chromium Next GEM Single Cell 3' v3.1 library preparation kit (10xGenomics) according to the manufacturer's protocol. The single-cell library was sequenced on Illumina NextSeq 550 instrument.

Processing of scRNA-seq data

Raw gene expression (UMI counts) matrix was generated using the standard Cell Ranger pipeline (v.4.0.0) from 10xGenomics (basecalling, mapping and counting) with the hg38 version of the human reference genome (as of July 7, 2020). Further data processing was done in R (version 3.6.3) using the Seurat package (v3.2.3).[2] This included initial filtering of low-quality cells and/or potential doublets, as well as removal of apoptotic cells (dying cells resulting from stress related to prior laboratory procedures). Specifically, cells expressing less than 500 or more than 5000 genes, cells with less than 1000 or more than 20000 UMIs, as well as cells with more than 10% mitochondrial RNA were removed. Additionally, genes not expressed in at least 10 cells were filtered out. After the quality control steps, the expression data of remaining cells were normalized using Seurat's *NormalizeData* function and the *LogNormalize* method and subsequently scaled using the *ScaleData* function, which included regressing out the UMI count and the percentage of mitochondrial RNA content. Principal component analysis based on the most variable genes was used to reduce the dimensionality of the data and after inspecting the elbow and JackStraw plots, we selected the first 20 PCs for subsequent unsupervised clustering of the data using the FindNeighbors and FindClusters functions. In the latter function, the resolution parameter was set to 0.6. Finally, the *RunUMAP* function with the same PCs as input to the clustering analysis was used for visualization to allow easy exploration of the data.

Identification of the immune cell types was done in a two-step process. First, the SingleR package (version 1.06),[3] which features automatic annotation method was used to label each single cell against the reference dataset *MonacoImmuneData* from Monaco et al. (2019) provided by the celldex package (version 0.99.1).[4] SingleR was run twice, first to return a more general cell type label (*label.main*) and second to yield fine-grained cell type classification (*label.fine*). The automatic cell type assignments were next verified by careful manual inspection of marker genes identified by the *FindAllMarkers* function and comparing the top genes (by log2 fold expression) in each cluster and cross-check with the canonical cell type markers known from the literature (Table S5).

Estimating mosaicism at single-cell level

Global and tissue type specific levels of mosaicism related to either the 45,X or the 48,XYYY status of cells were estimated based on the expression of Y-linked genes. No threshold but a simple requirement that all genes from Y identified in the sequenced data set are either expressed or not was used to perform the classification. The analysis excluded genes encoded by the Y pseudoautosomal regions (PARs) due to high homology of this short region to the X chromosome counterpart and because only the X chromosome versions of genes from the pseudoautosomal regions are present in the reference genome used by Cell Ranger. To further validate the above-described

methodology and its findings, we used other data processing tools. Alevin, a lightweight-mapping pipeline for scRNA-seq data pre-processing,[5,6] was used following similar assumptions in the data filtering steps and calling aneuploidy status. The software was run with default settings, additionally specifying the --*chromiumV3* flag and the --*tgMap* parameter pointing to the 'transcript to gene map' file generated for the Gencode v35 protein - coding genes only (https://www.gencodegenes.org/human/release_35.html). UMI-tools was used as a second independent validation tool.[7] We followed the standard steps described in the single cell tutorial (https://github.com/CGATOxford/UMI-tools). Additionally, we tested the level of potential read dropout due to non-unique mapping between the X-transposed MSY genes and their counterparts from chromosome X (MSY genes with X homologs). Reads without unique mapping to the reference genome receive low mapping scores and are not counted during the expression (UMI) matrix generation. Original read mapping from the STAR software [8,9] as returned by the Cell Ranger pipeline in the form of a BAM file was inspected to estimate the proportion of unique to non-unique mapping reads (reads with score 255 versus those with lower scores). An independent BWA alignment was constructed and analyzed in similar fashion.[10] Monte Carlo permutation test was used to check whether the observed differences in distribution of a given cell type between 45,X and 48,XYYY cells are significant and are not an effect of random sampling, with FDR value <0.05 used as statistically significant.

Comparison against public PBMC data

Three public single cell PBMC datasets were selected for comparison under a requirement of being generated with the same scRNA-seq technology, having the cell labels already assigned and representing male samples. GEO dataset *GSE132065* was filtered to keep only the healthy male controls and only the samples that were preserved immediately (0 hours).[11] Another PBMC datasets from healthy male donors were obtained from GEO dataset *GSE150728* and *GSE128879*.[12,13] In the case of *GSE128879*, we kept only the control samples that were not treated with LPS. All predefined cell labels available for the three datasets were cross-checked with the ones obtained from automatic labelling using SingleR package with the same reference dataset as used for the studied boy's cell.

Differential expression analyses

In order to identify genes that are significantly differentially expressed (DEG) between the 45,X and 48,XYYY type cells, statistical testing on the expression values was performed using the *FindMarkers* function in Seurat. The default Wilcoxon test was applied within each identified cell type separately and the resultant DEGs were filtered using a minimum log fold change of 0.2 and Bonferroni-adjusted p-value <0.05. Further filtering excluded all genes from the Y chromosome as well as genes from the chromosome X PAR region.

Supplementary Results

Detecting Y-linked expression and estimating the level of mosaicism

Droplet digital PCR (ddPCR) analysis of the FACS-sorted PBMC fractions clearly confirmed the presence of imbalance in the ratio of the mosaic cell populations. To verify whether such imbalance is reflected in the scRNA-seq data, we first inspected the expression patterns of the ubiquitously expressed genes from the male specific region of the Y chromosome (MSY) in all sequenced cells.[14,15] Overall, 19 genes were present in the data set, among which 10 protein-coding genes from MSY were found (Table S6). *RPS4Y1* was the most ubiquitously expressed gene present in nearly 50% of the cells in the dataset. Subsequently the status of each individual cell, either 45,X or 48,XYYY, was determined by the complete absence or presence of expression from these genes. We assumed that total lack of Y-linked expression is due to the actual 45,X karyotype of cells, since our data was sequenced to a very high depth (Table S2) to effectively recover even the lowly expressed genes. In the total single cell PBMC data, the 48,XYYY and 45,X cells constituted 48.43% and 51.57%, respectively (Figure 2C and Table S7). Independent analyses confirmed these results (Figure S5). Additional test for potential read dropout due to non-unique read mapping between the Y and X homologs of the MSY genes further validated the results (data not shown). This nearly perfect balance between the 45,X and 48,XYYY cells is no longer observed when looking into individual immune cell fractions. The majority of different T-cell populations contained Y, while B cells or monocytes were mostly composed of cells without the Y chromosome (Figure 2C and Table S7). The above observations match the ones from ddPCR results of FACS sorted cell fractions based on the *AMELY/AMELX* ratio (Figure 2D and Table S8). Although overall there was no significant difference in the number of expressed genes or UMIs in cells belonging to both populations, we saw that the high frequencies of triple Y cells were accompanied with significantly higher expression from Y chromosome (Figure 2E and Figure S6). This observation holds true also when compared to PBMCs of healthy male controls (Figure 2E and Figure S7).

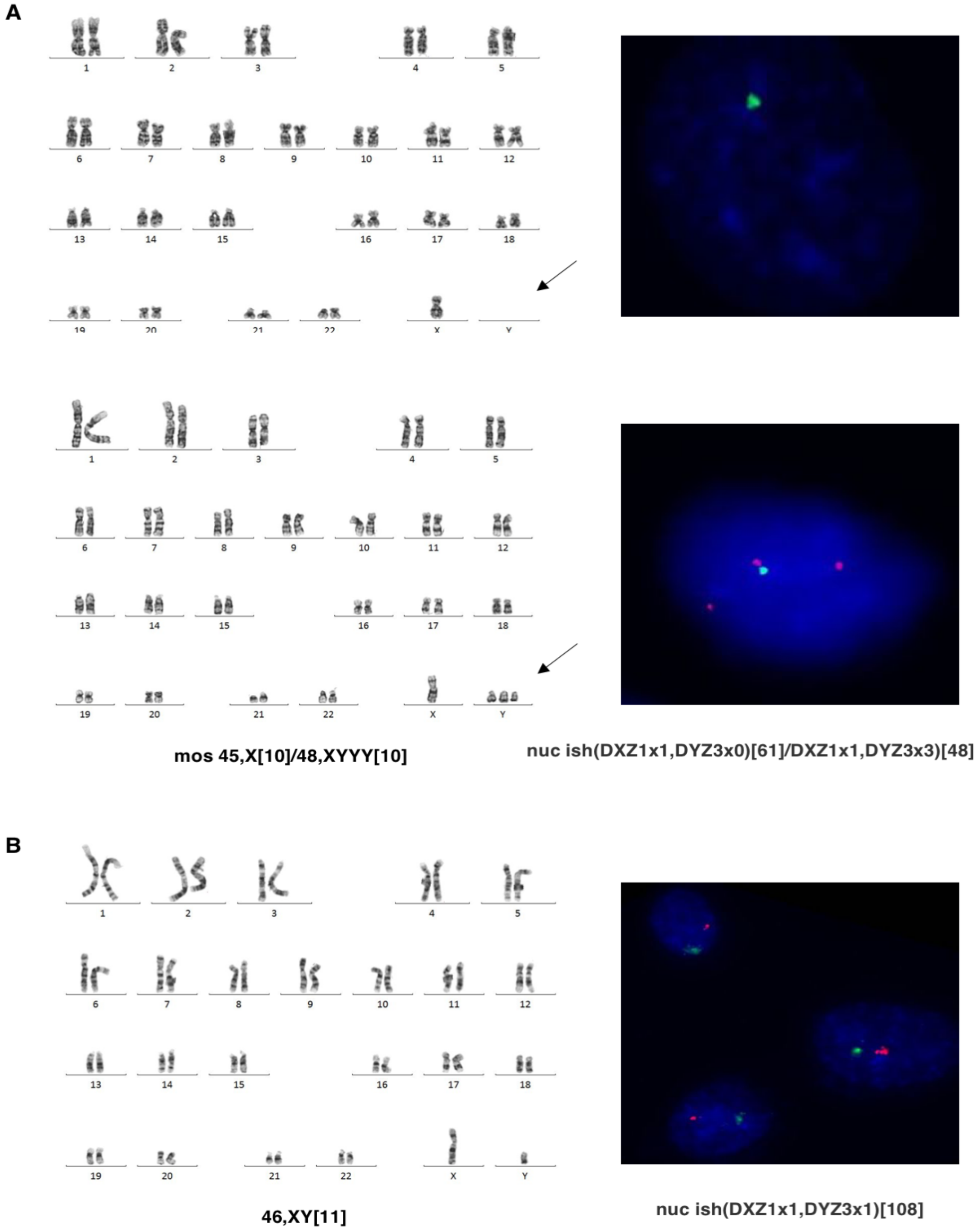

Figure S1. Cytogenetics results from fibroblast cultures.

Classical GTG-banding karyotyping at the 550-band stage of resolution and fluorescent *in situ* hybridization (FISH) on fibroblast cultures collected from the proband (**panel A**) and his father (**panel B**) were performed. The loss of chromosome Y and the triple chromosome Y are indicated by the arrows. The green and red signals are from chromosomes X and Y, respectively.

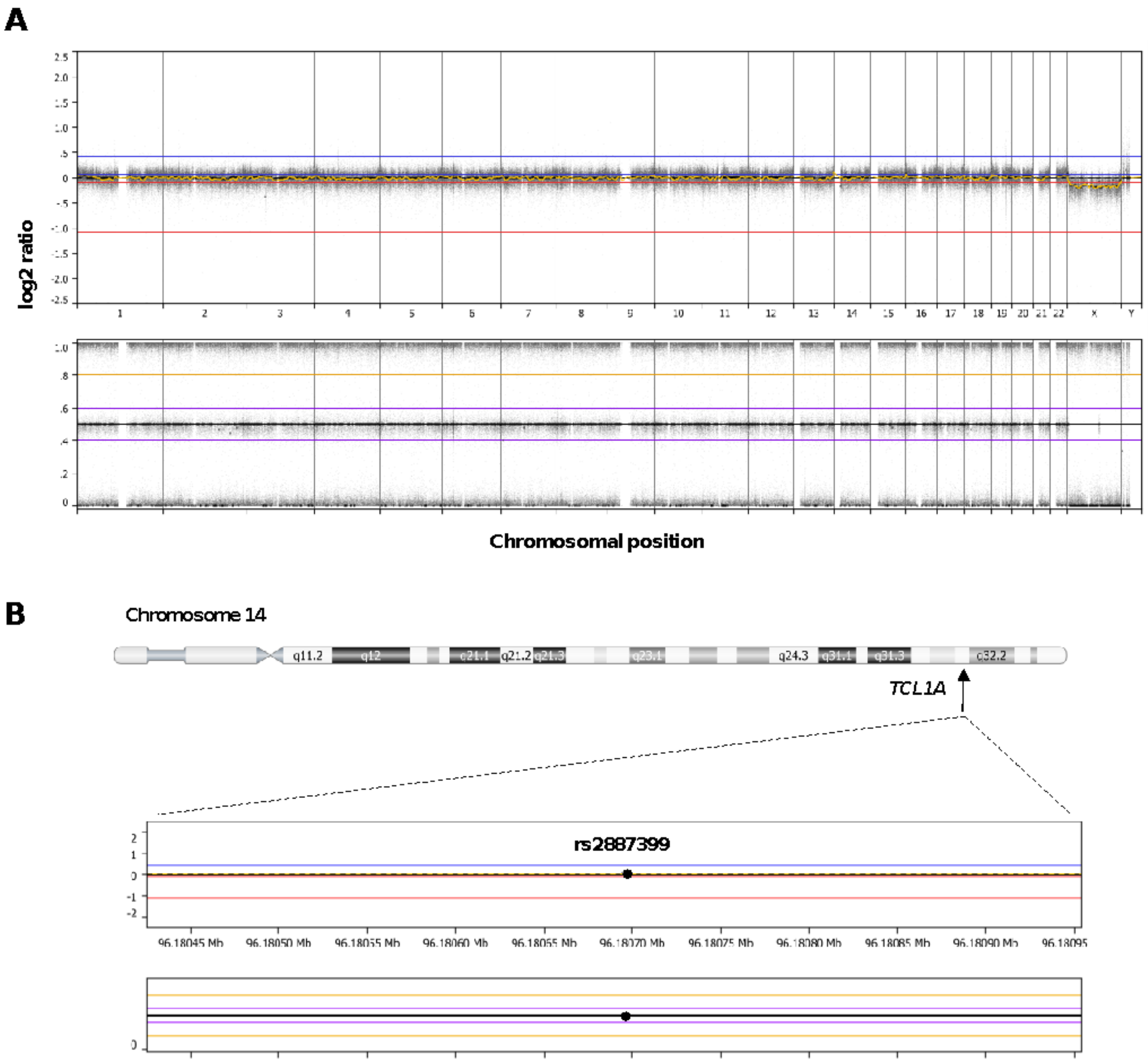

Figure S2. Chromosomal microarray analysis (CMA) results with the *TCL1A* SNP (rs2887399) locus.

Panel A shows an ideogram summarising no copy number aberrations in the patient's peripheral's blood, except for a loss of chromosome X and extra copies of chromosome Y. **Panel B** shows the specific location (chr14:96,180,696 bp) of the SNP (rs2887399) near the *TCL1A* gene.

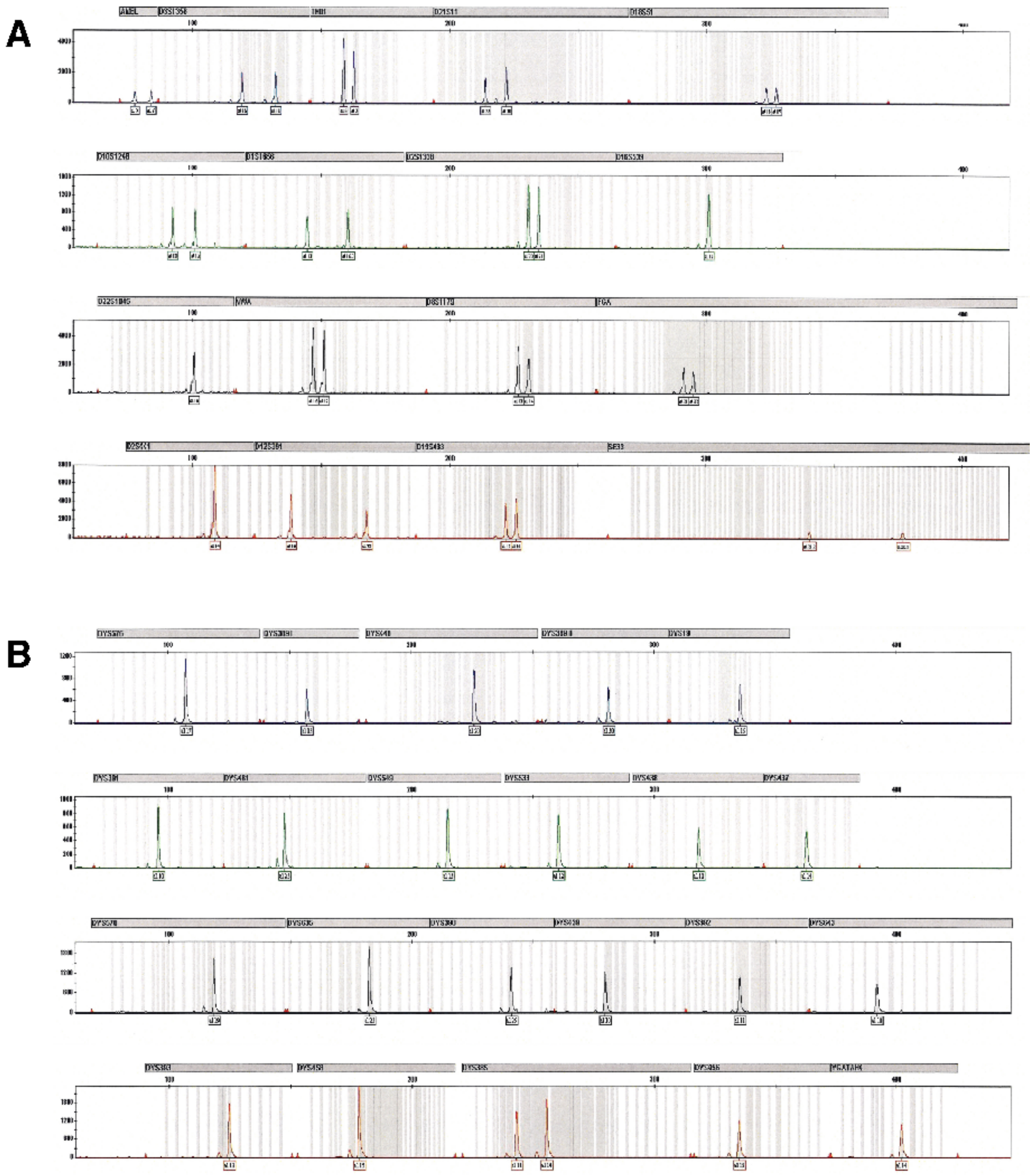

Figure S3. Autosomal and Y-chromosome short-tandem repeat (Y-STR) DNA typing results.

Panel A shows autosomal loci analysis using using PowerPlex® ES17 Fast kit (Promega) for 16 autosomal polymorphic loci and Amelogenin used as a sex marker. **Panel B** shows Y-STR analysis using PowerPlex® Y23 Systems (Promega) for 23 Y chromosome polymorphic loci.

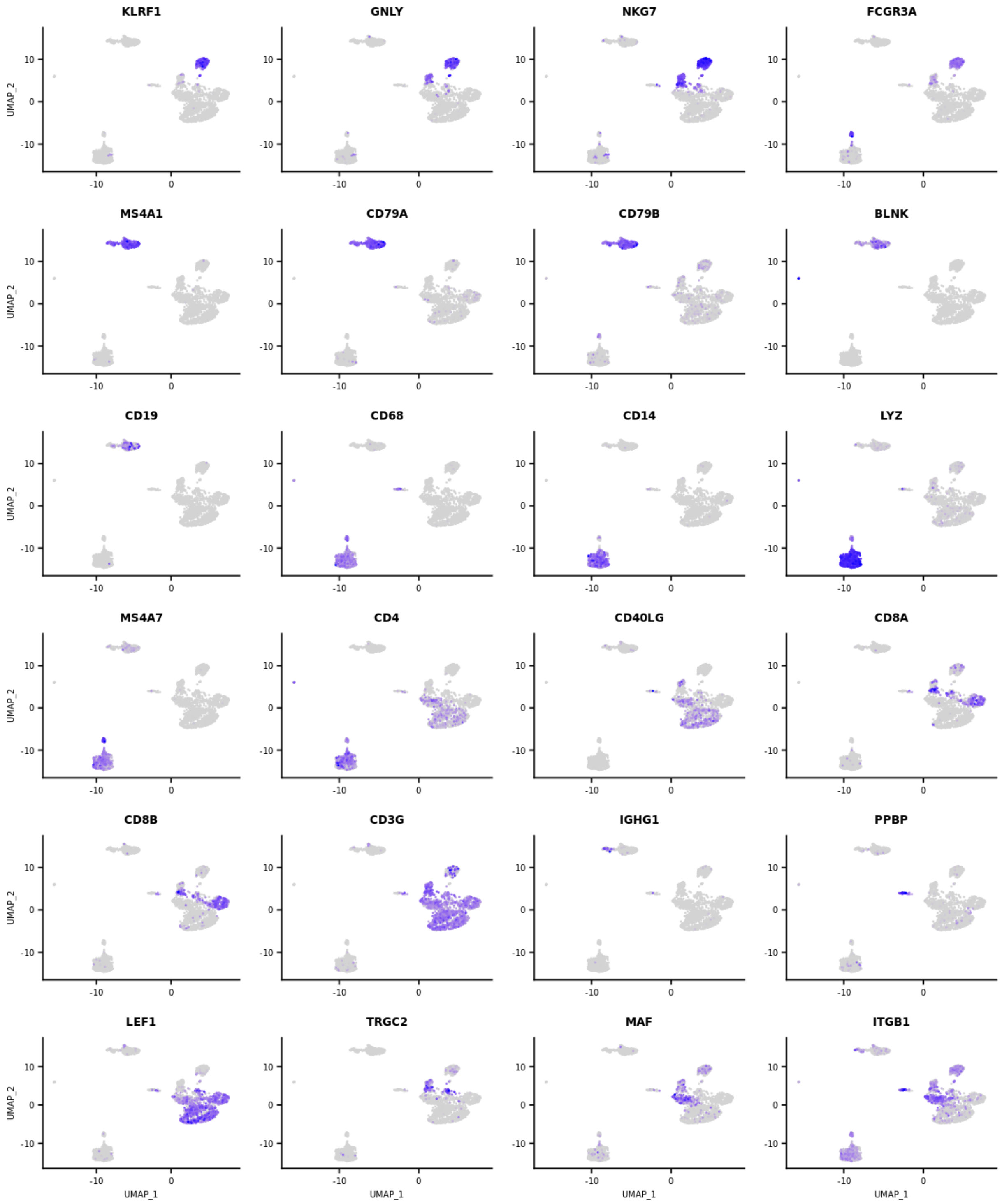

Figure S4. Selected marker genes used to verify the computationally inferred cell clusters.

Well-known genes representative to the major PBMC immune cell subtypes were selected from the Monaco et al. reference set [4] and their expression projected onto the two-dimensional UMAP clustering of cells. Blue color indicates activity of the gene, while grey corresponds to the lack of such. The darker the blue, the higher the gene expression level. Full list of marker genes and accompanying references is available in Table S5.

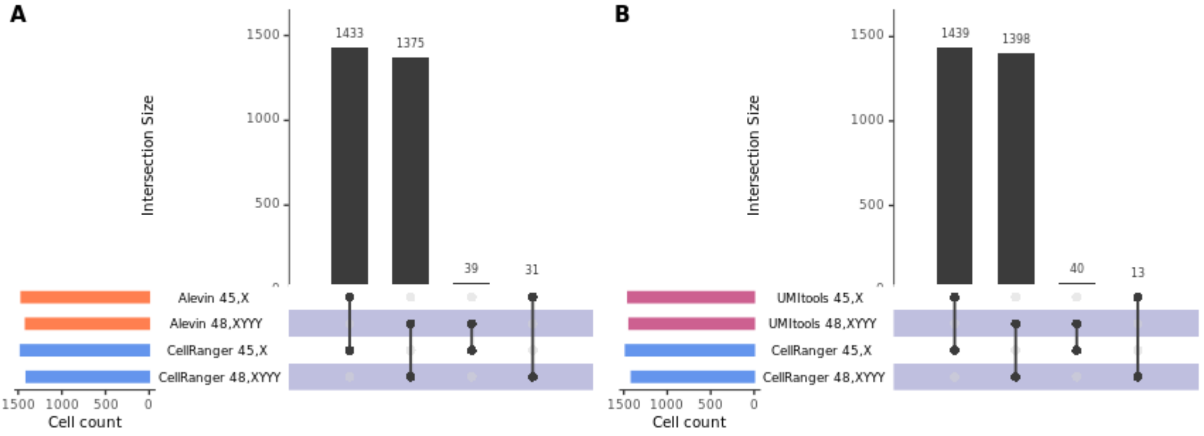

Figure S5. Separation of the PBMC single cells into the two mosaic populations 45,X and 48,XYYY verified by two independent methods for processing UMI based single cell data.

Panel A shows assignment of cells based on the presence or absence of the Y-linked expression conducted with Alevin matches the one obtained for CellRanger based data. Only a very small fraction of cells has a contradicting assignment. **Panel B** shows assignment of cells based on the presence or absence of the Y-linked expression conducted with UMItools matches the one obtained for CellRanger based data. Only a very small fraction of cells has a contradicting assignment. Vertical and horizontal bars show the number of cells identified by each tool and assigned to one of the mosaic cells fractions. Black dots connected with solid lines indicate intersecting sets.

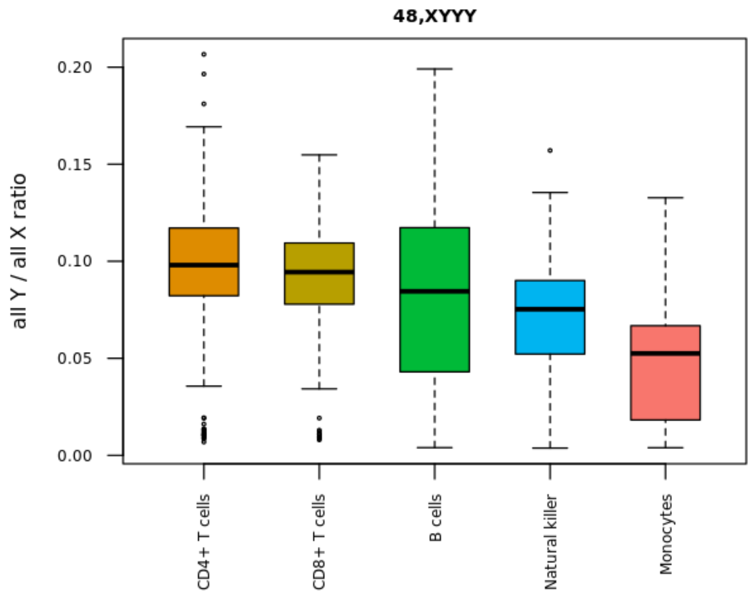

Figure S6. Ratios of Y-linked to X-linked expression (normalized) in the 48,XYYY cell population in selected fractions of the immune cell types.

The share of the Y-linked expression in the cells' transcriptome varies between the identified cell types with the highest levels observed in CD4+ and CD8+ T cells, while the lowest share is seen in monocytes.

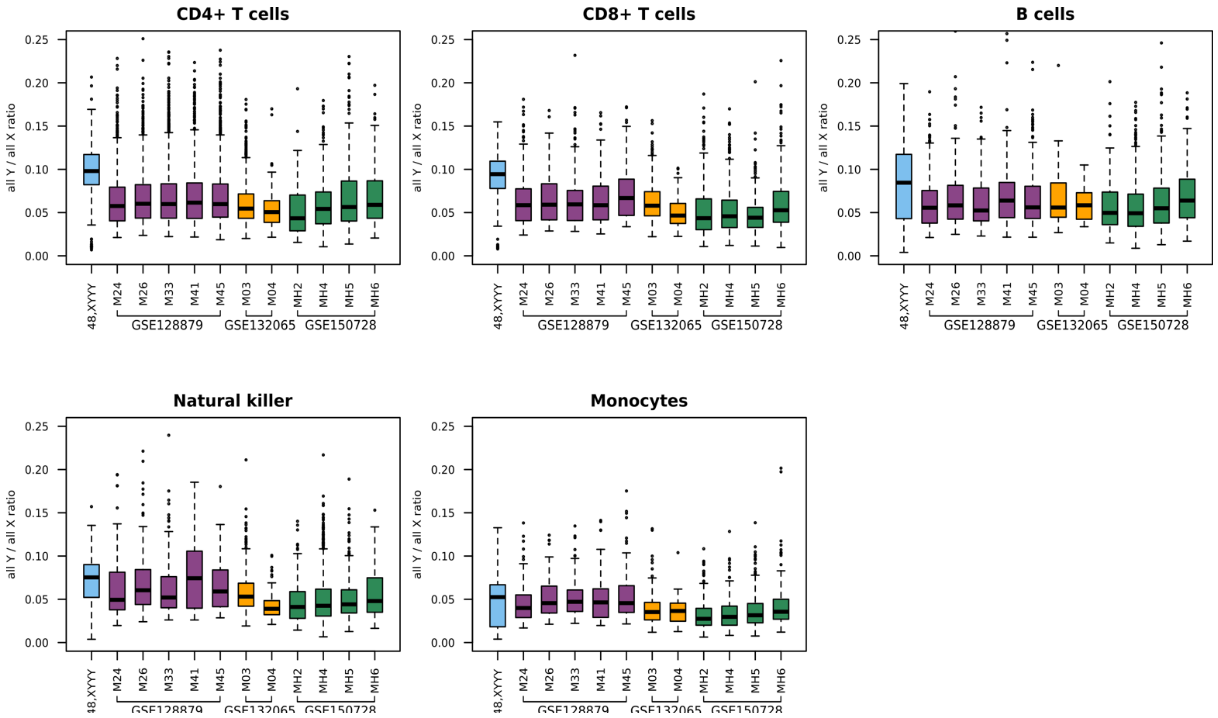

Figure S7. Ratios of the Y-linked to X-linked expression (normalized) in the 48,XYYY cell population as compared to the public PBMC data of healthy males.

Comparison is done for five selected fractions of the immune cell types, each plotted onto a separate panel. Three public datasets used for comparison, namely GSE128879 (Karagiannis et al. 2020), GSE132065 (Massoni-Badosa et al. 2020) and GSE150728 (Wilk et al. 2020), consist of five, two and four male samples, respectively.[11-13] Labels on the X axis correspond to the IDs of donors used in the public datasets or their age (GSE128879). In each of the panels the 48,XYYY fraction of the studied boy's PBMC displays a much higher Y/X ratio than the males used for comparison. All male cells were pre-filtered to discard all cells without any Y-linked expression to match the processing scheme of the PBMC of the studied boy yielding the 48,XYYY population.

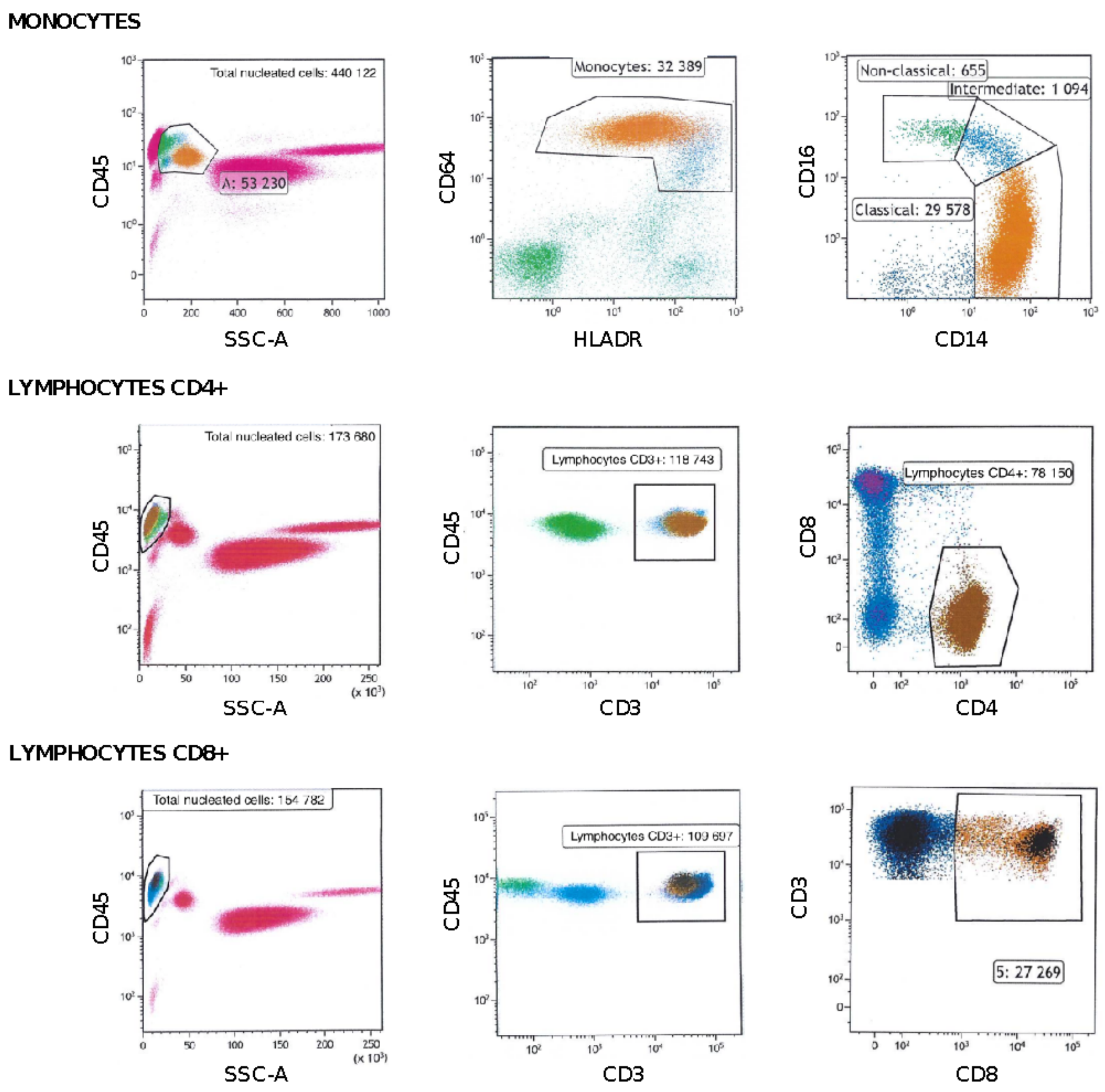

Figure S8. Flow cytometry immunophenotyping of monocytes and T lymphocytes in the blood of a proband as compared to a healthy control.

Table S1. Clinical features of 14 cases reported as with pure or mosaic 48,XYYY karyotype (as of July 2021).

Reference	Constitutional karyotype	Age (years)	Clinical presentation	Comments
Townes et al. (1965)[16]	48,XYYY[27]	5	Uncomplicated pregnancy and delivery; mild upper-respiratory infection with cardiac murmur at 2 weeks old (further cardiac evaluation at 3½ years old revealed an isolated pulmonary stenosis); mild psychomotor delay (walked independently at age 21 months, spoke first words at 2 years and simple sentences at 3 years);<br>clinical evaluation: 110.5 cm, dental dysplasia with brownish teeth, undescended testis, simian lines, pulmonary valve stenosis, left inguinal hernia, frequent upper-respiratory infections, slight hyperextensibility in knees and elbow, IQ 80 with mild speech delay, no other developmental abnormalities noted with normal neurological examination result.	Classical GTG-banding karyotyping with chromatin-negative buccal smear; the individual's healthy non-consanguineous parents (mother's age 29 and father's age 42) were reported to have normal karyotypes; the individual's healthy two sisters (age 6 and 3 years) and three brothers (age 7 years, 2 years and 2 months) were apparently healthy and not tested; negative family history, except for a female first cousin (a daughter of a maternal aunt) with a mosaic trisomy 21.
Cox and Berry (1967)[17]	mos 45,X[60]/ 48,XYYY[15]	4	Uncomplicated pregnancy and delivery; newborn diagnosed with short curved phallus with scrotal hypospadias and undescended testis;<br>clinical evaluation: DSD (disorder of sex development) individual with hypospadias,<br>a uterus with bindly-ending Fallopian tubes and a single testis on the right side, urinary steroid levels within normal limits, apparently normal intelligence, height and weight at the 6th percentile (the individual's parents were both short), no other developmental abnormalities noted.	Classical GTG-banding karyotyping with chromatin-negative buccal smear; the individual's healthy non-consanguineous parents (mother's age 24 and father's age 26) were reported to have normal karyotypes; the individual's older healthy sister was not tested.
Schoepflin and Centerwall (1972)[18]	48,XYYY[50]	9	Uncomplicated pregnancy (mother's age 27 and father's age 27) and delivery (Caesarean section after 38-week gestation); at 5 weeks hospitalized with upper respiratory infection associated with choking spells and cyanosis (chest X-ray showed atelectasis in the right base); diagnosed twice with bronchopneumonia at the age of 4 months and 4 years; significant psychomotor delay (walked independently at 2 years, spoke first 2-3 words together at 3 years);<br>clinical evaluation: 140 cm (75th percentile), mild psychomotor delay (special class), bilateral clinodactyly of the 5th digits, hypoplastic middle phalanges, modified simian crease on both hands, normal genitalia except for a small hydrocele on the left, urinary steroid levels within normal limits, delayed bone formation, IQ 79, no other developmental abnormalities noted with normal cardiac evaluation results.	Classical GTG-banding karyotyping of three different tissues: peripheral blood lymphocytes, bone marrow aspirate 48,XYYY[5] and skin fibroblasts 48,XYYY[1]; fluorescent staining of interphase and metaphase chromosomes confirmed classical karyotyping results; the individual's healthy parents were reported to have normal karyotypes; the individual's apparently healthy two sisters (age 10 and 12 years) and brother (age 7 years) were reported to have normal karyotypes; negative family history, except for a male first cousin with a mental retardation (a son of maternal aunt), diagnosis unknown, no chromosome studies performed.

Reference	Constitutional karyotype	Age (years)	Clinical presentation	Comments
Hunter and Quaife (1973)[19]	48,XYYY[72]	52	No developmental abnormalities were found, except for infertility due to azoospermia (two separate semen analyses revealed no spermatozoa) and developmental lag for secondary sexual characteristics, normal cardiac evaluation and EEG, tall stature (180 cm), IQ 65, behavior disturbances without aggressiveness.	Classical GTG-banding karyotyping; the individual's brother was reported to have normal karyotypes, other's family members' history was not available.
Ridler et al. (1973)[20]	48,XYYY[50]	>18	Uncomplicated pregnancy and delivery; IQ 81, severe acne, small hypotrophic testes, sex hormones normal, no sexual interest, myopia, behavior disturbances with aggressiveness, recurrent bronchitis (up to the age of 4 years), CALMs, sparse axillary and pubic hair.	Classical GTG-banding karyotyping confirmed by fluorescent staining and chromatin-negative buccal smear; 48,XYYY[48] karyotype was also reported in fibroblasts.
Sele et al. (1975)[21]	mos 46,XY[15]/ 47,XYY[6]/ 48,XYYY[27]	15	No developmental abnormalities were found, except for clinodactyly of the 5th digits and hypertension with polyuria, 167 cm, obesity, IG 53, the individual's penis and testes were of normal size, urinary steroid and testosterone levels normal.	Classical GTG-banding karyotyping confirmed by fluorescent staining and chromatin-negative buccal smear.
Gigliani et al. (1980)[22]	mos 47,XYY(27%)/ 48,XYY(68%)/ 49,XYYY(4%)	9	Mild psychomotor delay in the first years of life, several dysmorphic features (low forehead, palpebral fissures slanted upwards and outwards, epicanthus, telangiectasia of the scleras, prominent upper lip, dental anomalies, micrognathia, large and low set ears, bilateral hypoplasia of the shoulder girdle, bilateral simian crease, both feet in valgus), no details regarding the individual's genital was available; at the age of 5 years the individual suffered from grand mal seizures (the individual's maternal aunt suffered from the same disease).	Classical GTG-, C- and Q-banding karyotyping; the individual's father was reported to have a normal karyotype.
Hori et al. (1988)[23]	48,XYYY	29	Uncomplicated pregnancy and delivery (mother's age 29);<br>clinical evaluation: tall stature (183 cm), lack of sexual desire, CALMs, acne, incurved little fingers at both hands, small brownish teeth, short penis, incomplete phimosis, hypoplastic testes, azoospermia (testicular biopsy revealed atrophic seminiferous tubules containing only Sertoli cells with absence of spermatogenesis), high LH and FSH levels, low testosterone level, low borderline intelligence.	Classical GTG- and Q-banding karyotyping followed by fluorescent staining; the individual's parents were reported to have normal karyotypes; the individual's older healthy sister not tested; negative family history.
Bryke et al. (1989)[24]	mos 45,X[7]/ 48,XYYY[92]	prenatal	Pregnancy termination at 20-week gestation (mother's age 34 and father's age 41); fetus was reported to have ambiguous external genitalia, a horseshoe kidney, a cerebral cortical cyst, arachnodactyly (also present in the mother) and a camptodactyly suspicion, but no clinodactyly, autopsy revealed presence of intra abdominal gonads likely testes, no cardiac abnormalities noted.	Classical GTG-banding karyotyping was performed; non-consanguineous parents were reported to have normal karyotypes; the first child (the father's daughter from previous marriage) was apparently healthy, but not tested.

Reference	Constitutional karyotype	Age (years)	Clinical presentation	Comments
Mazaric-Stüker et al. (1992)[25]	48,XYYY[30]	33	Epileptic seizures, tall stature (185 cm) and leptosome habitus, hollow feet, onychocryptosis of the toes, slight spinal ataxia of the right side without neurological features, radioulnar synostosis at left elbow, congenital malformation of the right elbow, mental retardation with anamnesis difficulty (alcohol abuse), Morbus Fahr, light gynaecomastia without testicular hypotrophy, parathyroid and thyroid hormones within normal limits.	Classical Q- and C-banding karyotyping; the individual's healthy mother and a younger sister (age 25) not tested.
Teyssier and Pousset (1994)[26]	mos 46,XY([24]/ 48,XYYY[103]	37	Born prematurely at 8-month of gestation with neonatal asphyxia required intubation and artificial respiration; no developmental delay (walked independently at age 19 months and spoke at 2 years);<br>clinical evaluation: obesity (167 cm and 108 kg), dysplasia of the right hip (total prosthesis), IQ 65, behavior disturbances (aggressiveness with depressive periods and sexual impulsions), moderately hypoplastic testes with hydrocele on the left, testicular biopsy revealed fibrohyalinization in about 10% of the tubules and subnormal spermatogenesis in the others.	Classical GTG-banding karyotyping on two different tissues: peripheral blood lymphocytes and skin fibroblasts mos 46,XY[1]/47,XYY[1]/48,XYYY[128];<br>the individual's parents were not tested.
Fox et al. (1995)[27]	mos 45,X[20]/ 47,XYY[15]/ 48,XYYY[17]	< 2	Uncomplicated pregnancy and delivery (Cesarean section at 38-week gestation); ambiguous genitalia, minor ear and eye anomalies, coarctation of the aorta with bicuspid aortic valve, bilateral Fallopian tubes, left infantile testis with epididymi	Classical GTG-banding karyotyping, followed by FISH analysis on three additional tissues:<br>- aorta mos 45,X[45]/47,XYY[11]/48,XYYY[3],<br>- skin fibroblasts 45,X[75],<br>- Fallopian tube mos 45,X[76]/47,XYY[17]/48,XYYY[7<br>- testis mos 45,X[57]/47,XYY[39]/48,XYYY[4].
Venkataraman and Craft (2002)[28]	48,XYYY[25]	prenatal	Pregnancy through ICSI treatment (mother's age 35) terminated at 18-week gestation; parents were reported to have normal karyotypes.	Classical GTG-banding karyotyping of different tissues: kidney, lung and gut, all with a 48,XYYY[25] karyotype.
Abedi et al. (2017)[29]	48,XYYY[81]	32	Uncomplicated pregnancy and delivery; clinical evaluation: tall stature (190 cm), teeth dysmorphology, history of frequent upper-respiratory infections, arachnodactyly, partial deformity of the joints and nails, infertility.	Classical GTG-banding karyotyping, followed by QF-PCR.

Abbreviations: DSD - disorder of sex development; EEG - electroencephalogram; CALMs - café-au-lait macules; LH - luteinizing hormone; FSH - follicle stimulating hormone; mos - mosaic; QF-PCR - quantitative fluorescent polymerase chain reaction; ICSI - intracytoplasmic sperm injection.

Table S2. Summary of high throughput sequencing and Cell Ranger results.

Raw reads	611 492 256
Reads assigned to sequencing sample	582 693 629
Sequencing saturation	91.5%
Estimated number of cells	3 337
Mean reads per cell	174 616
Median UMI counts per cell	7 178
Median genes per cell	2 026
Total genes detected	22 933

Table S3. Summary of quality filtering steps within Seurat.

Number of cells before QC	3 337	100%
Number of cells with >10% of mitochondrial RNA	217	6.5%
Number of cells with <500 genes	147	4.41%
Number of cells with >5000 genes	17	0.51%
Number of cells with <1000 UMIs	99	2.97%
Number of cells with >20000 UMIs	52	1.56%
Final number of cells after quality check	2 936	**87.99%**
Total genes detected	22 933	100%
Number of genes expressed in ≥ 10 cells	15 779	**68.8%**

Table S4. Summary of clustering with Seurat.

**Cluster ID**	**Number of cells**	**Percentage of cells**	**Total number of genes**	**Median number of genes**	**Total UMIs**	**Median UMIs**
0	503	17.13	14762	3010	4592834	8948
1	456	15.53	14383	1889.5	3375304	7402
2	439	14.95	14334	1841	3253509	7372
3	363	12.36	14506	2098	3408269	8933
4	281	9.57	14228	1783	1774347	5624
5	278	9.47	14280	2287.5	2438384	8589.5
6	240	8.17	13885	2166.5	1410210	5620
7	213	7.25	13909	2156	1491570	6750
8	61	2.08	12173	2096	539552	7940
9	60	2.04	12109	3461	752062	13201
10	33	1.12	5544	742	101732	2245
11	9	0.31	7149	2138	70579	7899

Table S5. Clusters calculated with Seurat by applying the default Louvain algorithm and their labels (main or fine grained) assigned with SingleR using the Monaco et al. reference set.[4] The last column represents arbitrarily selected literature-based marker genes used to validate the automatic labels.

Cluster	Main label	Fine grained label	Selected marker genes
0	Monocytes	Classical monocytes	CD68+, CD14+, CD68+, LYZ+, FCGR3A (CD16)-
9	Monocytes	Non-classical monocytes	CD69+, CD14+, CD68+, LYZ+, FCGR3A (CD16)+
1, 2	CD4+ T cells	Naïve CD4 T cells	CD4+, CD40LG+, CD3G+, LEF1+, CD8A-, CD8B-, MAF-
5	CD4+ T cells	Follicular helper T cells / T regulatory cells	CD4+, CD40LG+, CD3G+, LEF1+, CD8A-, CD8B-, MAF+
3	CD8+ T cells	Naïve CD8 T cells	CD8A+, CD8B+, ITGB1-, MAF-, TRGC2-
7	T cells	Vδ2 T cells / non-Vδ2 T cells	CD8A+, CD8B+, ITGB1+, MAF+, TRGC2+
4	B cells	Naïve B cells	MS4A1+, MS4A7+, CD79A+, CD79B+, BLNK+, CD19+, IGHG1+
8	B cells	Switched / non-switched memory B cells	MS4A1+, MS4A7+, CD79A+, CD79B+, BLNK+, CD19+, IGHG1-
6	Natural killer	Natural killer	KLRF1+, GLNY+, NKG7+
10	Progenitor cells	Progenitor cells	PPBP+

Table S6. Genes from chromosome Y identified in the studied sample.

Symbol	Description	Gene type	Cells number	Cells (%)	Total UMIs
RPS4Y1	ribosomal protein S4 Y-linked 1	protein coding	1454	49.52	21229
EIF1AY	eukaryotic translation initiation factor 1A Y-linked	protein coding	954	32.49	1930
USP9Y	ubiquitin specific peptidase 9 Y-linked	protein coding	891	30.35	1792
DDX3Y	DEAD-box helicase 3 Y-linked	protein coding	855	29.12	1516
UTY	ubiquitously transcribed tetratricopeptide repeat containing, Y-linked	protein coding	857	29.19	1411
PRKY	protein kinase Y-linked (pseudogene)	protein coding	689	23.47	1171
ZFY	zinc finger protein Y-linked	protein coding	463	15.77	642
KDM5D	lysine demethylase 5D	protein coding	464	15.80	627
LINC00278	long intergenic non-protein coding RNA 278	lncRNA	351	11.96	483
TTTY14	testis-specific transcript, Y-linked 14	lncRNA	227	7.73	286
AC244213.1	novel transcript, antisense to USP9Y	lncRNA	165	5.62	192
TTTY10	testis-specific transcript, Y-linked 10	lncRNA	85	2.90	93
AC006157.1	novel transcript	lncRNA	51	1.74	52
TMSB4Y	thymosin beta 4 Y-linked	protein coding	45	1.53	45
AC010889.1	novel transcript	lncRNA	28	0.95	29
AC010889.2	novel transcript, antisense to KDM5D	lncRNA	19	0.65	20
RPS4Y2	ribosomal protein S4 Y-linked 2	protein coding	17	0.58	20
ZFY-AS1	ZFY antisense RNA 1	lncRNA	16	0.54	19
LINC00266-4P	long intergenic non-protein coding RNA 266-4 (pseudogene)	lncRNA	12	0.41	12

Table S7. Summary statistics of 45,X and 48,XYYY cells identified among individual clusters and the entire sample.

Cluster ID	Cells with Y present	Cells with Y present (%)	Cells without Y	Cells without Y (%)
0	163	32.41	340	67.59
1	441	96.71	15	3.29
2	40	9.11	399	90.89
3	199	54.82	164	45.18
4	75	26.69	206	73.31
5	204	73.38	74	26.62
6	103	42.92	137	57.08
7	154	72.3	59	27.7
8	23	37.7	38	62.3
9	10	16.67	50	83.33
10	3	9.09	30	90.91
11	7	77.78	2	22.22
**Total**	1422	48.43	1514	51.57

Table S8. Results of digital droplet PCR (ddPCR) assay.

	**1st Run**				**2nd Run**					
**Cell type**	***AMELY* conc. (copies/L)**	***AMELX* conc. (copies/L)**	**Accepted Droplets**	**Ratio 1 (Y/X)**	***AMELY* conc. (copies/L)**	***AMELX* conc. (copies/L)**	**Accepted Droplets**	**Ratio 2 (Y/X)**	**Ratio Mean (Y/X)**	**SD**
PBMC	135	109	12620	1.25	148	114	14086	1.29	1.27	0.02
non-Treg	1705	1113	14619	1.53	1328	842	15922	1.58	1.5545	0.0225
Treg	761	441	18336	1.73	697	410	17267	1.70	1.715	0.015
CD19+	183	363	17124	0.51	199	383	17305	0.52	0.5115	0.0065
CD8+	880	561	16266	1.57	760	490	16552	1.55	1.56	0.01
Monocytes	387	540	17389	0.72	413	568	16826	0.73	0.7215	0.0055
Natural Killer	290	302	17368	0.96	279	295	17265	0.95	0.953	0.007
Granulocytes	299	441	16505	0.65	342	518	15851	0.66	0.65675	0.00325
Buccal swab	647	376	15512	1.72	490	285	15957	1.72	1.72	0
Whole Blood	425	455	14737	0.94	266	303	17224	0.88	0.9065	0.0285

Abbreviations: PBMC - peripheral blood mononuclear cells; SD - standard deviation.

Table S9. List of 31 differentially expressed genes between 45,X and 48,XYYY populations.

Gene	Gene type	Name	Upregulated cluster	p_val	avg_logFC	pct.1	pct.2	p_val_adj
***TCL1A* ***	protein coding	TCL1 family AKT coactivator A	45,X B cells	0	0.5697472	0.951	0.853	0.0000022
NOSIP	protein coding	nitric oxide synthase-interacting protein	45,X CD4+ T cells	0	0.3689621	0.903	0.794	0
CHI3L2	protein coding	chitinase 3 like 2	45,X CD4+ T cells	0	0.3642697	0.391	0.175	0
STMN1	protein coding	stathmin 1	45,X CD4+ T cells	0	0.2965518	0.478	0.318	0.0001839
ITM2A	protein coding	integral membrane protein 2A	45,X CD4+ T cells	0.0000007	0.2919845	0.71	0.603	0.0104058
RPL23	protein coding	ribosomal protein L23	45,X CD4+ T cells	0	0.2651354	0.995	1	0
PAX8-AS1	lncRNA	PAX8 antisense RNA 1	45,X TFH/TREG	0	0.2844618	0.257	0.039	0.0006415
			45,X Monocytes	0	0.3859027	0.4	0.11	0.0000006
			45,X CD4+ T cells	0	0.3127542	0.239	0.025	0
OVCH1-AS1	lncRNA	OVCH1 antisense RNA 1	45,X TFH/TREG	0	0.2599722	0.216	0	0.0000001
SNHG25	lncRNA	small nucleolar RNA host gene 25	48,XYYY CD4+ T cells	0.0000001	0.2411621	0.699	0.582	0.0008959
			48,XYYY CD8+ T cells	0	0.3675704	0.804	0.579	0.0003462
S100A4	protein coding	S100 calcium binding protein A4	48,XYYY B cells	0	0.6431786	0.507	0.136	0.0000021
			48,XYYY CD4+ T cells	0	0.6591246	0.574	0.297	0
GSTP1	protein coding	glutathione S-transferase pi 1	48,XYYY TFH/TREG	0	0.5390214	0.657	0.27	0.0000557
			48,XYYY CD8+ T cells	0	0.374446	0.633	0.341	0.0000459
			48,XYYY CD4+ T cells	0	0.3908315	0.541	0.242	0
CRIP1	protein coding	cysteine rich protein 1	48,XYYY B cells	0.0000012	0.6158383	0.467	0.199	0.0189051
***ANXA1* ***	protein coding	annexin A1	48,XYYY CD4+ T cells	0	0.4202611	0.462	0.159	0
S100A11	protein coding	S100 calcium binding protein A11	48,XYYY CD4+ T cells	0	0.3226096	0.543	0.353	0.0000032
CD48	protein coding	CD48 molecule	48,XYYY CD4+ T cells	0	0.2815717	0.969	0.93	0
BST2	protein coding	bone marrow stromal cell antigen 2	48,XYYY CD4+ T cells	0	0.2778647	0.435	0.234	0.0000016
GPR183	protein coding	G protein-coupled receptor 183	48,XYYY CD4+ T cells	0	0.2746386	0.484	0.278	0.0000106
CAPN2	protein coding	calpain 2	48,XYYY CD4+ T cells	0	0.2363829	0.212	0.036	0
FXYD5	protein coding	FXYD domain containing ion transport regulator 5	48,XYYY CD4+ T cells	0	0.2289517	0.89	0.79	0.0001525
PPIB	protein coding	peptidylprolyl isomerase B	48,XYYY CD4+ T cells	0	0.2178846	0.78	0.63	0.000635
NAP1L1	protein coding	nucleosome assembly protein 1 like 1	48,XYYY CD4+ T cells	0.0000001	0.214673	0.909	0.862	0.0016983
PYURF	protein coding	PIGY upstream reading frame	48,XYYY CD4+ T cells	0.0000001	0.2114674	0.565	0.389	0.0020126
MIF	protein coding	macrophage migration inhibitory factor	48,XYYY CD4+ T cells	0	0.2095731	0.954	0.886	0.0000081
GAPDH	protein coding	glyceraldehyde-3-phosphate dehydrogenase	48,XYYY CD4+ T cells	0.0000016	0.2076633	0.879	0.831	0.0248956
AHNAK	protein coding	AHNAK nucleoprotein	48,XYYY CD4+ T cells	0.0000018	0.2022396	0.202	0.092	0.0289433
KLRB1	protein coding	killer cell lectin like receptor B1	48,XYYY TFH/TREG	0.0000008	0.9842112	0.475	0.162	0.0121482
ITGA4	protein coding	integrin subunit alpha 4	48,XYYY TFH/TREG	0.0000001	0.6080091	0.76	0.378	0.0013127
TRGC1	TR_C_gene	T-cell receptor gamma chain constant region 1	48,XYYY Vδ2 T cells	0.0000011	0.9093216	0.643	0.322	0.0173481
IL7R	protein coding	interleukin 7 receptor	48,XYYY Vδ2 T cells	0.0000002	0.8336571	0.864	0.627	0.0037447
HOPX	protein coding	HOP homeobox	48,XYYY Vδ2 T cells	0	0.7723986	0.675	0.22	0.0000468
TPT1	protein coding	tumor protein, translationally-controlled 1	48,XYYY Vδ2 T cells	0.0000008	0.2483421	1	1	0.0121737

* The dysregulated expression of these genes was also confirmed by an independent study by Dumanski et al. (2021),[30] i.e. the *ANXA1* expression was downregulated in 45,X T cells, while the *TCL1A* expression was upregulated in 45,X B cells.

Table S10. Description of selected genes potentially related to cancer/hematological complications.

Gene (MIM)	Description
upregulated in 45,X	
STMN1 **(*151442)**	A stathmin, a microtubule destabilizing protein important for the function of the mitotic spindle, is expressed strongly in the cells of various types of human acute leukemia;[31] gene overexpression has been associated with cancer progression and chemoresistance in several cancer types, i.e. lung cancer, gastric cancer or breast cancer.[32-34]
TCL1A **(*186960)**	The protein acts as a co-activator of the cell survival kinase AKT enhancing cell proliferation and survival; the overexpression is usually observed in hematological malignancies of T and B cells;[35] chromosomal rearrangements, including inversions or translocations involving the *TCL1A* gene are observed in more than 90% of T-cell prolymphocytic leukemias.
upregulated in 48,XYYY	
AHNAK **(*103390)**	The gene encodes scaffold protein that participates in calcium signalling in T cells and is a binding partner of Formin-like 1 (FMNL1), highly expressed in hematopoietic cells and leukemias.[36-38]
ANXA1 **(*151690)**	The protein has an anti-inflammatory effect, i.e. AnxA1 deficiency significantly increased antigen-induced T cell proliferation and the resulting inflammation;[39] besides, it has been related with cancer progression and metastasis likely by activating PI3K/AKT signaling pathway with a potential regulating role in tumor cell proliferation.[40-41]
BST2 **(*600534)**	Bone marrow stromal cell antigen 2 (CD317) is a multiple myeloma-associated antigen that is over-expressed in B-cell chronic lymphocytic leukemia and might be considered as its prognostic marker;[42] their role in the regulation of B cell activation remain unknown, however, recent study shows that it might be involved as a BAFF-responsive membrane factor in the regulation of NF-κB signaling.[43]
CAPN2 **(*114230)**	The calpains (calcium-activated neutral proteases), encoded by the *CAPN2* gene, represent non lysosomal intracellular cysteine proteases with the presumed function in T-cell receptor signaling by cytoskeletal remodeling;[44] besides, overexpression of CAPN2 promotes cell metastasis and proliferation via AKT/mTOR signaling pathway in several cancer types.[45-46]
CRIP1 **(*123875)**	Cysteine rich protein 1 is characterized by restricted expression in normal tissue, but activated in tumors and thus considered as a general marker of cancer; for instance, in cervical cancer this protein promotes cell migration, invasion and epithelial-mesenchymal transition of cancer cells by activating the Wnt/β-catenin signaling pathway.[47]
EZR **(*123900)**	This protein is expressed strongly in certain human tumors, including B-cell lymphoma, in which it has been reported as a novel regulator of pathogenic B-cell receptor signaling and tumor growth.[48]
GSTP1 **(*134660)**	The protein glutathione S-transferase belongs to a family of enzymes that play a crucial role in detoxification and have antioxidant properties. The decreased expression has been observed in various cancers, such as prostate or liver cancer.[49-50]
MIF **(*153620)**	Macrophage migration inhibitory factor (MIF) plays as oncogene by inducing angiogenesis;[51] an increased expression of the *MIF* has been frequently observed in solid tumors.[52]
LIMS1 **(*602567)**	The increased expression of *LIMS1* has been associated with advanced TNM stage and poor prognosis of cancer patients.[53]
S100A4 **(*114210)**	Metastasin, a protein encoded by *S100A*4 is a member of the S100 protein family, particularly associated with the progression and metastasis of numerous human malignancies; its role is described as a vital part of the T-cell machinery aimed against immunoevasive cells through performing both prometastatic and antimetastatic functions.[54]
TRGC1 **(*186970)**	Children with T-cell acute lymphoblastic leukemia expressing the gamma-delta T-cell receptor have been reported to have worse prognosis;[55] a subset of gamma-delta T-cells is overrepresented in the non-responding tumors (melanoma) to immune checkpoint therapy;[56] little is known about the molecular mechanisms leading to responses of gamma-delta T cells.

Table S11. Results of standard hematological analysis of the proband's peripheral blood.

Cell type		Percentage of cells	
		**Proband**	**Reference adjusted to age**
**Leukocytes**	**CD45+**	39.7	32 - 45
**T cells**	**CD3+**	69.9	68 - 74
**Suppressor T cells**	**CD3+/CD8+**	17.7 ↘	30 - 36
**Helper T cells**	**CD3+/CD4+**	45.3 ↗	30 - 36
**Naïve helper T cells**	**CD4+/CD45RA+**	74.9	21 - 75
**Memory helper T cells**	**CD4+/CD45RO+**	25.0	11 - 44
**Activated T cells**	**CD3+/HLA DR**	7.7	4 - 10
**Index**	**CD4 versus CD8**	2.6 ↗	1.0 - 1.5
**B cells**	**CD19+**	18.6	13 - 19
**Natural killer**	**CD16+/CD56+**	10.5	7 - 15

Table S12. Details of Monte Carlo permutation test.

Clusters	45,X	48,XYYY	obs_log2FD	pval	FDR	boot_mean_log2 FD	boot_CI_2.5	boot_CI_97.5
B cells	0.161162483	0.068917018	-1.2255838	0.000999001	0.001498501	-1.2242194	-1.56410264	-0.90940311
CD4+ T cells	0.273447820	0.338255977	0.3068499	0.000999001	0.001498501	0.3071943	0.15197527	0.47435543
CD8+ T cells	0.108322325	0.139943741	0.3695164	0.014985015	0.019266448	0.3665749	0.08249277	0.67867227
Monocytes	0.257595773	0.121659634	-1.0822583	0.000999001	0.001498501	-3.3646309	-5.03883928	-1.89467006
Natural killer	0.090488771	0.072433193	-0.3210878	0.042957043	0.048326673	-1.0811831	-1.32343038	-0.83155375
TFH/TREG	0.048877147	0.143459916	1.5534157	0.000999001	0.001498501	1.8069910	0.09044374	3.54987536
Vδ2 T cells	0.038969617	0.108298172	1.4745872	0.000999001	0.001498501	-0.3292341	-0.69384158	0.02173099

Table S13. Details of gene-set enrichment analysis.

ID	Description	Gene Ratio	Bg Ratio	p-value	p-adjust	q-value	gene ID	Count
GO:0070663	regulation of leukocyte proliferation	5/15	151/10917	0.0000013	0.0011757	0.0007018	*ANXA1/GSTP1/BST2/GPR183/MIF*	5
GO:0070661	leukocyte proliferation	5/15	198/10917	0.0000048	0.0021919	0.0013084	*ANXA1/GSTP1/BST2/GPR183/MIF*	5
GO:0001817	regulation of cytokine production	6/15	491/10917	0.0000284	0.0065585	0.0039151	*ANXA1/GSTP1/BST2/CAPN2/MIF/GAPDH*	6
GO:0060326	cell chemotaxis	4/15	150/10917	0.0000415	0.0076773	0.0045829	*ANXA1/GSTP1/GPR183/MIF*	4
GO:0033559	unsaturated fatty acid metabolic process	3/15	58/10917	0.0000619	0.0095305	0.0056892	*ANXA1/GSTP1/MIF*	3
GO:0001776	leukocyte homeostasis	3/15	65/10917	0.0000871	0.0098796	0.0058976	*ANXA1/GPR183/MIF*	3
GO:0050482	arachidonic acid secretion	2/15	11/10917	0.0000962	0.0098796	0.0058976	*ANXA1/MIF*	2
GO:1903963	arachidonate transport	2/15	11/10917	0.0000962	0.0098796	0.0058976	*ANXA1/MIF*	2

References

1. Danielsson M, Halvardson J, Davies H, et al. Longitudinal changes in the frequency of mosaic chromosome Y loss in peripheral blood cells of aging men varies profoundly between individuals. Eur J Hum Genet. 2020; 28:349-57.
2. Butler A, Hoffman P, Smibert P, Papalexi E, Satija R. Integrating single-cell transcriptomic data across different conditions, technologies, and species. Nat Biotechnol. 2020; 36: 411-20.
3. Aran D, Looney AP, Liu L, et al. Reference-based analysis of lung single-cell sequencing reveals a transitional profibrotic macrophage. Nat Immunol 2109; 20: 163-72.
4. Monaco G, Lee B, Xu W, et al. RNA-Seq signatures normalized by mRNA abundance allow absolute deconvolution of human immune cell types. Cell Reports 2019; 26: P1627-40.
5. Srivastava A, Malik L, Sarkar H, Patro R. A Bayesian framework for inter-cellular information sharing improves dscRNA-seq quantification. Bioinformatics 2020; 36: i292-9.
6. Srivastava A, Malik L, Smith T, Sudbery I, Patro R. Alevin efficiently estimates accurate gene abundances from dscRNA-seq data. Genome Biol. 2019; 20: 65.
7. Smith T, Heger A, Sudbery I. UMI-tools: modeling sequencing errors in Unique Molecular Identifiers to improve quantification accuracy. Genome Res. 2017; 27: 491-9.
8. Dobin A, Davis CA, Schlesinger F, et al. STAR: ultrafast universal RNA-seq aligner. Bioinformatics 2013; 29: 15-21.
9. Dobin A, Gingeras TR. Mapping RNA-seq Reads with STAR. Curr. Protoc. Bioinforma.2015. John Wiley & Sons, Inc., Hoboken, NJ, USA, pp. 11.14.1-11.14.19.
10. Li H, Durbin R. Fast and accurate short read alignment with Burrows-Wheeler transform. Bioinformatics 2009; 25: 1754-60.
11. Massoni-Badosa R, Iacono G, Moutinho C, et al. Sampling time-dependent artifacts in single-cell genomics studies. Genome Biol. 2020; 21: 112.
12. Wilk AJ, Rustagi A, Zhao NQ, et al. A single-cell atlas of the peripheral immune response in patients with severe COVID-19. Nat Med. 2020; 26: 1070-6.
13. Karagiannis TT, Cleary JP, Gok B, et al. Single cell transcriptomics reveals opioid usage evokes widespread suppression of antiviral gene program. Nat. Commun. 2020; 11: 2611.
14. Maan AA, Eales J, Akbarov A, et al. The Y chromosome: a blueprint for men's health? Eur J Hum Genet. 2017; 25: 1181-8.
15. Barros B, Morais M, Teixeira AL, Medeiros R. Loss of Chromosome Y and Its Potential Applications as Biomarker in Health and Forensic Sciences. Cytogenet Genome Res. 2020; 160: 225-37.
16. Townes PL, Ziegler NA, Lenhard LW. A patient with 48 chromosomes (XYYY). Lancet. 1965; 1: 1041-3.
17. Cox D, Berry CL. A patient with 45,XO - 48,XYYY mosaicism. J Med Genet. 1967; 4: 132-3.
18. Schoepflin GS, Centerwall WR. 48,XYYY: a new syndrome? J Med Genet. 1972; 9(3): 356-60.
19. Hunter H, Quaife R. A 48,XYYY male: a somatic and psychiatric description. J Med Genet. 1973: 10: 80-3.
20. Ridler MA, Lax R, Mitchell MJ, Shapiro A, Saldana- Garcia P. An adult male with XYYY sex chromosomes. Clin Genet. 1973; 4: 69-77.
21. Sele B, Bachelot Y, Richard J, Muller J, Jalbert P, Berthet J. [48,XYYY males. Apropos of a case of 46,XX/47,XYY/48,XYYY mosaicism] Pediatrie 1975; 30: 601-7 [article in French].
22. Gigliani F, Gabellini P, Petrinelli P, Antonelli A. Peculiar mosaicism 47,XYY/48,XYYY/49,XYYYY in man. J Genet Hum. 1980; 28: 47-51.
23. Hori N, Kato T, Sugimura Y, Tajima K, Tochigi H, Kawamura J. A male subject with 3 Y chromosomes (48,XYYY): a case report. J Urol. 1988; 139: 1059-61.
24. Bryke CR, Mahoney MJ, Yang-Feng TL. Antenatal diagnosis of 45,X/48,XYYY. Am J Med Genet. 1989; 34: 207-10.
25. Mazauric-Stüker M, Kordt G, Brodersen D. Y aneuploidy: a further case of a male patient with a 48,XYYY karyotype and literature review. Ann Genet. 1992; 35: 237-40.
26. Teyssier M, Pousset G. 46,XY/48,XYYY mosaicism case report and review of the literature. Genet Couns. 1994; 5: 357-61.
27. Fox JE, Blumenthal D, Brock W, et al. Infant with mos 45,X/46,XY/47,XYY/48,XYYY: genetic and clinical findings. Am J Med Genet. 1995; 59: 435-440.

28. Venkataraman G, Craft I. Triple-Y syndrome following ICSI treatment in a couple with normal chromosomes. Case report. Hum Reprod. 2002; 17: 2560-3.
29. Abedi M, Salmaninejad A, Sakhinia E. Rare 48,XYYY syndrome: case report and review of the literature. Clin Case Rep. 2017; 6: 179-84.
30. Dumanski J, Halvardson J, Davies H, et al. Immune cells lacking Y chromosome show dysregulation of autosomal gene expression. Cell Mol Life Sci. 2021; 78: 4019-33.
31. Machado-Neto JA, Saad ST, Traina F. Stathmin 1 in normal and malignant hematopoiesis. BMP Rep. 2014; 47: 660-5.
32. Bao P, Yokobori T, Altan B, et al. High STMN1 expression is associated with cancer progression and chemo-resistance in lung squamous cell carcinoma. Ann Surg Oncol. 2017; 24: 4017-24.
33. Bai T, Yokobori T, Altan B, et al. High STMN1 level is associated with chemo-resistance and poor prognosis in gastric cancer patients. Br J Cancer 116; 1177-85.
34. Askeland C, Wik E, Finne K, et al. Stathmin expression associates with vascular and immune responses in aggressive breast cancer subgroups. Sci Rep. 2020; 2914.
35. Laine J, Kunstle G, Obata T, Sha M, Noguchi M. The protooncogene TCL1 is an Akt kinase coactivator. Mol Cell. 2000; 6: 395-407.
36. Radermacher AN, Crabtree GR. Monster protein controls calcium entry and fights infection. Immunity. 2008; 28: 13-4.
37. Matza D, Flavell RA. Roles of Ca(v) channels and AHNAK1 in T cells: the beauty and the beast. Immunol. Rev. 2009; 231: 257-64.
38. Han Y, Yu G, Sarioglu H, Caballero-Martinez A, Schlott F, Ueffing M, Haase H, Peschel C, Krackhardt. Proteomic investigation of the interactome of FMNL1 in hematopoietic cells unveils a role in calcium-dependent membrane plasticity. J Proteomics. 2013; 78: 72-82.
39. Yang YH, Song W, Deane JA, Kao W, Ooi JD, Ngo D, Kitching AR, Morand EF, Hickey MJ. Deficiency of annexin A1 in the CD4+ T cells exacerbates T cell-dependent inflammation. J Immunol. 2013; 190: 997-1007.
40. Cardin LT, Prates J, Rodrigues da Cunha B, Tajara EH, Oliani SM, Rodrigues-Lisoni FC. Annexin A1 peptide and endothelial cell-conditioned medium modulate cervical tumorigenesis. FEBS Open Bio. 2019; 9: 668-81.
41. Zhu JF, Huang W, Yi HM, et al. Annexin A1-suppressed autophagy promotes nasopharyngeal carcinoma cell invasion and metastasis by PI3K/AKT signaling activation. Cell Death Dis 2018; 9: 1154.
42. Gong S, Osei ES, Kaplan D, Chen YH, Meyerson H. CD317 is over-expressed in B-cell chronic lymphocytic leukemia, but not B-cell acute lymphoblastic leukemia. Int J Clin Exp Pathol. 2015; 8: 1613-21.
43. Fu J, Shi H, Zhan T, Li H, Ye L, Xie L, Wang Z, Wang B, Zheng L. BST-2/Tetherin is involved in BAFF-enhanced proliferation and survival via canonical NF-κB signaling in neoplastic B-lymphoid cells. Exp Cell Res. 2021; 398: 112399.
44. Morford LA, Forrest K, Logan B, Overstreet K, Goebel J, Brooks WH, Roszman TL. Calpain II colocalizes with detergent-insoluble rafts on human and Jurkat T-cells. Biochem Biophys Res Commun. 2002; 295: 540-6.
45. Libertini SJ, Robinson BS, Dhillon NK, Glick D, George M, Dandekar S, Gregg JP, Sawai E, Mudryj M. Cyclin E both regulates and is regulated by calpain 2, a protease associated with metastatic breast cancer phenotype. Cancer Res. 2005; 65: 10700-8.
46. Miao C, Liang C, Tian Y, et al. Overexpression of CAPN2 promotes cell metastasis and proliferation via AKT/mTOR signaling in renal cell carcinoma. Oncotarget 2017; 8: 97811-21.
47. Zhang LZ, Huang LY, Huang AL, Liu JX, Yang F. CRIP1 promotes cell migration, invasion and epithelial-mesenchymal transition of cervical cancer by activating the Wnt/β-catenin signaling pathway. Life Sci. 2018; 207: 420-7.
48. Pore D, Bodo J, Danda A, Yan D, Phillips JG, Lindner D, Hill BT, Smith MR, Hsi ED, Gupta N. Identification of Ezrin-Radixin-Moesin proteins as novel regulators of pathogenic B-cell receptor signaling and tumor growth in diffuse large B-cell lymphoma. Leukemia 2015; 29: 1857-67.
49. Varisli L. Identification of new genes downregulated in prostate cancer and investigation of their effects on prognosis. Genet Test Mol Biomarkers. 2013; 17: 562-6.

50. Li T, Zhao XP, Wang LY, Gao S, Zhao J, Fan YC, Wang K. Glutathione S-transferase P1 correlated with oxidative stress in hepatocellular carcinoma. Int J Med Sci. 2013; 10: 683-90.
51. Xu X, Wang B, Ye C, Yao C, Lin Y, Huang X, Zhang Y, Wang S. Overexpression of macrophage migration inhibitory factor induces angiogenesis in human breast cancer. Cancer Lett. 2008; 261: 147-57.
52. Lue H, Thiele M, Franz J, Dahl E, Speckgens S, Leng L, Fingerle-Rowson G, Bucala R, Luscher B, Bernhagen J. Macrophage migration inhibitory factor (MIF) promotes cell survival by activation of the Akt pathway and role for CSN5/JAB1 in the control of autocrine MIF activity. Oncogene 2007; 26: 5046-59.
53. Hung C, Li Y, Li Z, et al. LIMS1 promotes pancreatic cancer cell survival under oxygen-glucose deprivation conditions by enhancing HIF1A protein translation. Clin Cancer Res. 2019; 25: 4091-4103.
54. Donato R, Cannon BR, Sorci G, Riuzzi F, Hsu K, Weber DJ, Geczy CL. Functions of S100 proteins. Curr Mol Met. 2013; 13: 24-57.
55. Pui C, Pei D, Cheng C, et al. Treatment response and outcome of children with T-cell acute lymphoblastic leukemia expressing the gamma-delta T-cell receptor. Oncoimmunology. 2019; 8: 1599637.
56. Xiong D, Wang Y, You M. A gene expression signature of TREM2^{hi} macrophages and γδ T cells predicts immunotherapy response. Nat Commun. 2020; 11: 5084.
